# Correlation-Induced Accessibility Bridges in Biomedical Networks: A Proof-of-Concept Relational Graph Model

**DOI:** 10.3390/e28070769

**Published:** 2026-07-07

**Authors:** Roxana Irina Iancu, Călin Gheorghe Buzea, Florin Nedeff, Diana Mirilă, Valentin Nedeff, Mirela Panainte-Lehaduș, Claudia Manuela Tomozei, Maricel Agop, Alina Ștefania Doboș, Dragoş Petru Teodor Iancu, Lăcrămioara Ochiuz, Decebal Vasincu

**Affiliations:** 1 Department of Oral Pathology, “Gr. T. Popa” University of Medicine and Pharmacy, 700115 Iaşi, Romania; roxana.iancu@umfiasi.ro; 2 Department of Oncology and Radiotherapy, “Gr. T. Popa” University of Medicine and Pharmacy, 700115 Iaşi, Romania; 3 Department of Clinical Laboratory, “Sfântul Spiridon” Emergency Hospital, 700111 Iași, Romania; 4 National Institute of Research and Development for Technical Physics—IFT Iași, 700050 Iași, Romania; calinb2003@yahoo.com; 5 Clinical Emergency Hospital “Prof. Dr. Nicolae Oblu” Iași, 700309 Iași, Romania; 6 Department of Environmental Engineering, Mechanical Engineering and Agritourism, Faculty of Engineering, “Vasile Alecsandri” University of Bacău, 600115 Bacău, Romania; florin_nedeff@ub.ro (F.N.); vnedeff@ub.ro (V.N.); mirelap@ub.ro (M.P.-L.); claudia.tomozei@ub.ro (C.M.T.);; 7 Faculty of Physics, Alexandru Ioan Cuza University of Iași, 700506 Iași, Romania; alina_stefania.dobos@yahoo.com; 8 Department of Radiotherapy, Regional Institute of Oncology, 700483 Iași, Romania; 9 Faculty of Medicine, “Grigore T. Popa” University of Medicine and Pharmacy Iași, 700115 Iași, Romania; lacramioara.ochiuz@umfiasi.ro (L.O.); decebal.vasincu@umfiasi.ro (D.V.)

**Keywords:** biomedical networks, disease connectivity, relational graph model, accessibility bridges, mutual information, radiomics, systems medicine, tumor progression, multimodal prognosis, graph-based modeling, correlation-induced accessibility, disease-state geometry

## Abstract

Complex diseases often involve distributed interactions among biological regions, physiological systems, imaging phenotypes, and clinical variables that are not fully captured by anatomical proximity, isolated biomarkers, or conventional feature-based representations. In oncology, neuroimaging, critical care, and systems medicine, distant or apparently separate biomedical sectors may show strong statistical or functional coupling associated with multimodal imaging signatures, inflammatory responses, metabolic constraints, treatment-induced changes, or shared disease-state organization. In this work, we introduce a proof-of-concept relational graph framework for representing such candidate hidden connectivity in terms of correlation-induced accessibility bridges. The novelty of the framework is that it does not treat biomedical correlation, graph distance, and network connectivity as separate descriptors but explicitly couples non-factorizable inter-sector correlation to localized accessibility compression in an emergent disease-state geometry. The proposed framework represents a biomedical system as a weighted relational graph in which nodes correspond to clinically relevant entities, such as tissue regions, imaging-derived features, biomarker modules, physiological variables, or disease states, while weighted edges encode constraints on functional, statistical, or pathological accessibility. Within this structure, coarse-grained biomedical sectors are defined as organized subsystems, and non-factorizable coupling between sectors is quantified using mutual-information-type measures. Candidate biomedical bridges are then defined operationally as localized, high-gain reductions in effective inter-sector accessibility distance. We introduce explicit coupling rules linking sector-level correlation to bridge-specific accessibility compression, including an effective distance-compression model and an ensemble-based formulation. Numerical proof-of-concept simulations on randomized modular graph ensembles show that increasing correlation strength systematically reduces effective inter-sector distance and increases bridge gain. The strongest compression occurs when correlation modulates a designated bridge architecture, exceeding the effects observed under random non-bridge or generic inter-sector modulation. These simulations are not intended to validate a disease-specific biological mechanism but to test whether the proposed correlation–compression rule produces bridge-specific effects distinguishable from null graph perturbations. The resulting structures should not be interpreted as physical anatomical tunnels or direct causal pathways unless supported by additional biological evidence. Rather, they represent correlation-induced accessibility bridges: localized, high-gain routes in a patient- or disease-specific relational geometry. The framework may therefore provide a theoretical and computational basis for prioritizing candidate hidden connectivity patterns in radiomics, multimodal prognosis, physiological deterioration, recurrence modeling, and systems-level disease networks.

## 1. Introduction

Complex diseases are increasingly understood not as isolated local abnormalities but as emergent states of interacting biological, physiological, imaging, and clinical systems. In oncology, neuroimaging, critical care, cardiometabolic disease, and systems medicine, pathological behavior often depends on relationships among multiple variables or tissue compartments rather than on a single dominant lesion or biomarker. Tumor progression, metastatic dissemination, treatment response, organ failure, neurological deterioration, and recurrence may all involve coordinated changes across regions or domains that are not necessarily adjacent in anatomical space. This has motivated a shift from purely local descriptions of disease toward network-based, information-theoretic, and multiscale representations of biomedical organization [1,2,3,4,5,6,7,8,9].

Support for this perspective comes from systems biology, network medicine, graph-based modeling, radiomics, and machine learning approaches to multimodal medical data [5,6,7,9,10,11,12,13]. In these settings, biological function and disease progression may be represented through networks of interacting genes, proteins, imaging features, clinical variables, physiological measurements, or tissue compartments [1,3,14]. Disease modules, biomarker clusters, organ-system interactions, and imaging phenotypes can be reconstructed from patterns of statistical dependence, functional association, or shared pathological behavior [2,3,15]. Closely related ideas appear in radiomics and quantitative imaging, where spatially distributed features from tumor regions, peritumoral tissue, edema, necrosis, perfusion maps, or functional images may reveal disease structure not captured by conventional anatomical assessment alone [4,10,11,12,13]. In parallel, multimodal clinical prediction, temporal electronic-health-record graph modeling, and integrated biomedical AI frameworks have shown that disease-relevant information may emerge from dependencies among heterogeneous clinical, imaging, physiological, and temporal variables rather than from isolated biomarkers alone [16,17,18]. Together, these developments suggest that clinically meaningful disease connectivity may be representational and emergent rather than reducible to direct anatomical contiguity.

Despite these advances, many biomedical models still rely on predefined anatomical regions, fixed causal assumptions, or feature spaces that do not explicitly distinguish between ordinary correlation and structurally meaningful disease connectivity. Network medicine often begins from known molecular or physiological interaction maps; radiomics typically extracts high-dimensional features from defined regions of interest; and clinical machine-learning models frequently identify predictive associations without reconstructing the effective accessibility structure through which disease-relevant sectors may become functionally connected [19,20,21,22,23]. Even when graph methods are used, the resulting edges may represent statistical association, physical adjacency, or empirical similarity without a clear operational interpretation as reduced disease-state distance. This raises an important methodological question: can hidden biomedical connectivity be formulated in a framework where effective disease geometry is not assumed in advance but emerges from constrained accessibility among clinically relevant variables, tissues, or biological sectors?

The novelty of the present work lies in formulating hidden biomedical connectivity as a correlation-induced accessibility-compression problem. Existing biomedical network approaches often represent connectivity through known biological interactions, anatomical adjacency, feature similarity, statistical association, or predictive dependence. By contrast, the present framework focuses on the specific case in which a strong non-factorizable correlation between two biomedical sectors is represented as a localized reduction in effective accessibility distance. The proposed approach therefore does not merely construct a graph from biomedical variables, compute mutual information, or analyze shortest-path distances separately. Instead, it links these elements through an explicit bridge mechanism, in which bridge gain serves as an operational observable for distinguishing bridge-specific compression from generic inter-sector connectivity. In this way, the paper introduces a graph-theoretical formulation by which strongly coupled biomedical sectors may become effectively close in disease-state geometry without assuming anatomical continuity or direct causal pathways.

This positioning distinguishes the proposed framework from several related approaches. To clarify this positioning, Table 1 summarizes the main similarities and differences between the proposed accessibility-bridge framework and related biomedical graph, network, radiomics, information-theoretic, and graph-learning approaches.

Thus, the proposed framework should be viewed as complementary to existing biomedical graph methods. Its specific contribution is not graph construction alone, mutual information estimation alone, or predictive graph learning alone, but the definition of a localized accessibility-compression mechanism linking non-factorizable dependence to bridge-like disease-state proximity.

In network medicine, connectivity is commonly derived from known molecular, cellular, or physiological interaction maps; here, by contrast, effective connectivity is inferred through accessibility compression between coarse-grained biomedical sectors. In radiomics and quantitative imaging, graph or feature-based models often emphasize spatial organization, texture similarity, or predictive imaging signatures; the present framework instead asks whether such sector-level dependence can be represented as a bridge-like reduction in disease-state distance. In clinical machine-learning models, associations among variables may improve prediction without necessarily providing a structural interpretation of why distinct domains become jointly informative; the proposed bridge gain offers a graph-theoretical observable for this type of hidden coupling. Finally, while information-theoretic approaches quantify dependence through entropy- or mutual-information-type measures, the present model connects such dependence to an explicit accessibility geometry. Thus, the contribution is not the replacement of existing biomedical network methods but the introduction of a minimal relational mechanism linking non-factorizable correlation to localized accessibility compression.

A relational–informational graph framework provides a natural setting in which to address this question. In this framework, the primitive structure is a weighted network of biomedical or informational degrees of freedom connected by admissible informational relations, each subject to quantifiable constraints on accessibility or flow. A detailed foundational formulation was developed previously [24]. Effective geometry is defined operationally through minimal constraint cost, yielding an emergent accessibility metric without assuming spatial embedding. Dynamics are introduced through local changes in relational configurations, and a statistical description of these configurations gives rise to effective forces and information-geometric structure at the coarse-grained level [24]. Within this architecture, geometry is not ontological but representational: it is an effective encoding of constrained relational accessibility. For biomedical applications, this means that anatomical distance, feature similarity, functional dependence, and disease-state proximity can be treated as distinct but interacting layers of a more general accessibility structure.

This framework is suitable for reformulating hidden disease connectivity because effective biomedical proximity is not restricted to anatomical adjacency. If accessibility is derived from constrained relational organization, then strongly coupled sectors may become effectively close even when they are physically distant, belong to different modalities, or represent different biological scales. A tumor region and a peritumoral region, a biomarker module and an imaging phenotype, a respiratory variable and a neurological outcome, or a primary tumor and a metastatic niche may therefore be linked by an accessibility bridge if their relational coupling lowers the effective cost of transition, shared pathological expression, or predictive dependence.

The aim of the present work is to develop a correlation-induced accessibility-bridge model within a biomedical relational graph setting. More specifically, we ask whether two sufficiently coupled biomedical sectors—or two stable disease-relevant motifs—can become connected by an emergent low-cost bridge in the accessibility metric, thereby generating a candidate hidden route of disease connectivity without assuming direct anatomical continuity. To address this question, we introduce a mutual-information-type measure of non-factorizable biomedical correlation between subsystems, define observables of effective inter-sector accessibility, and study how graph-based disease configurations change when correlation strength and bridge cost are coupled. The central hypothesis is that a strong biomedical correlation may be encoded geometrically as anomalous compression of effective disease-state distance.

The purpose of the present paper is not to claim that correlation alone proves causality, nor that graph-based bridges automatically correspond to anatomical channels, vascular routes, neural tracts, metastatic pathways, or mechanistic biological links. Such interpretations require independent validation through clinical, imaging, molecular, longitudinal, or experimental evidence. Rather, the goal is methodological: to identify minimal structural conditions under which non-factorizable biomedical correlation and bridge-like accessibility compression can emerge as dual effective descriptions within a relational graph substrate.

The specific contribution of the present paper is therefore threefold. First, we give an operational definition of a biomedical accessibility bridge as a localized, stable, high-gain reduction in effective distance in a weighted biomedical relational graph. Second, we define sector-level biomedical coupling as robust, non-factorizable coarse-grained correlation quantified by a mutual-information-type observable, without assuming that such correlation is necessarily causal or mechanistic by itself. Third, we introduce explicit coupling rules and graph-ensemble simulations showing that correlation-dependent bridge modulation produces stronger accessibility compression than several null models. The result is not a direct clinical prediction model, but a constructive mathematical and computational framework for studying correlation–connectivity relations in biomedical systems where effective disease geometry is inferred rather than assumed.

To keep the construction focused, the present manuscript is restricted to the minimal definitions, coupling rules, and proof-of-concept simulations needed to establish this correlation–accessibility correspondence.

The paper is organized as follows. Section 2 recalls the minimal relational-graph substrate and adapts its notation to biomedical systems. Section 3 introduces mutual-information-type correlation measures between coarse-grained biomedical sectors. Section 4 defines accessibility-bridge observables and bridge gain criteria. Section 5 couples biomedical correlation to accessibility compression through phenomenological and ensemble-based rules. Section 6 presents the proof-of-concept graph simulation protocol. Section 7 reports the representative numerical results. Section 8 discusses interpretation, limitations, scaling, generalizability, and future directions toward radiomics, multimodal prognosis, recurrence modeling, and patient-specific disease networks. Section 9 provides the conclusions.

## 2. Biomedical Relational Substrate and Coarse-Grained Accessibility

The purpose of this section is to restate, in minimal form, the relational–informational framework required for the present biomedical extension. Our goal is not to reproduce the full foundational development given previously but to isolate the structures needed to formulate a correlation-induced accessibility-bridge model in complex medical systems. In particular, we require: (i) a biomedical relational substrate, (ii) an emergent notion of disease-state or functional distance derived from constrained accessibility, (iii) a statistical description over admissible biomedical configurations, and (iv) a definition of coarse-grained sectors suitable for expressing non-factorizable biomedical correlation and bridge-like disease connectivity. These ingredients are adapted here to the narrower purposes of computational medicine, radiomics, and systems-level disease modeling.

### 2.1. Biomedical Relational Configurations and Constraint Structure

We begin from the same general relational postulate but reinterpret it in biomedical terms. The primitive structure of the present model is a network of clinically relevant entities linked by admissible biomedical relations subject to quantifiable constraints, rather than a purely anatomical map, fixed causal diagram, or predefined feature space. These entities may represent imaging features, tissue regions, tumor compartments, organs at risk, biomarker modules, physiological variables, molecular pathways, treatment-response states, or clinically defined disease states.

Formally, a biomedical relational configuration is represented by a weighted graph

(1) $$G=\left(V,E,C\right)$$

where *V* is a set of nodes representing biomedical degrees of freedom, *E* ⊆ *V* × *V* is a set of admissible relations, and

(2) $$C:E\to R_{\geq 0}$$

assigns to each relation a nonnegative constraint weight. The quantity *C*(*e*) is interpreted operationally as the cost, resistance, limitation, or difficulty associated with biomedical accessibility along edge e. Depending on the application, this cost may encode anatomical separation, physiological resistance, statistical dissimilarity, weak functional coupling, radiomic incompatibility, molecular pathway distance, reduced information flow, or low probability of pathological transition.

Because the meaning of nodes and edge costs depends on the biomedical context, Table 2 provides representative examples of how the relational graph can be instantiated in different medical modeling settings [5,6,7,9,10,11,12,13].

To make the clinical use of Table 2 operational, the transition from a biomedical problem to the proposed graph model can be organized as a clinical-to-graph translation workflow. First, the clinical question should be defined explicitly, for example, diagnosis, prognosis, recurrence risk, treatment response, physiological deterioration, or patient stratification. Then, the relevant biomedical sectors should be selected, such as tumor core, peritumoral edema, organ-system compartments, biomarker modules, imaging domains, or ICU physiological domains. Furthermore, graph nodes should be assigned to clinically measurable entities, including imaging features, tissue regions, laboratory biomarkers, physiological variables, molecular pathways, or disease-state descriptors. Fourth, edge costs should be defined according to the application, for example, feature dissimilarity, weak statistical dependence, transition difficulty, pathway separation, reduced information flow, or low probability of coordinated state transition.

Moreover, inter-sector dependence should be estimated using a mutual-information-type quantity T(A:B), with uncertainty assessment and, where appropriate, background adjustment. Then, candidate accessibility bridges should be identified by testing whether specific edge sets or relay structures produce localized reductions in effective inter-sector distance and increased bridge gain. Furthermore, bridge candidates should be compared against null controls, such as permutation-based correlation nulls, degree- or weight-preserving graph rewiring, random non-bridge modulation, or random inter-sector modulation. Finally, any bridge-derived quantity should be validated against independent clinical endpoints, longitudinal outcomes, biological evidence, or external datasets. Until such validation is performed, the resulting accessibility bridges should be reported as hypothesis-generating structural candidates, not as direct causal mechanisms, diagnostic biomarkers, treatment rules, or clinical decision criteria.

No single biomedical interpretation is imposed at this level. In a radiomics study, nodes may correspond to imaging features or spatial tumor subregions, and edge weights may encode feature dissimilarity or reduced co-variation. In an ICU setting, nodes may correspond to physiological variables, laboratory values, blood-gas parameters, oxygenation indices, or clinical outcomes, while edges may encode constrained statistical dependence or transition difficulty. In oncology, nodes may correspond to primary tumor features, peritumoral regions, metastatic niches, immune components, or treatment-response phenotypes. The graph therefore encodes admissible biomedical accessibility and its associated constraints, rather than assuming that disease connectivity is reducible to physical adjacency alone.

This relational starting point is essential for the present work. Many clinically important interactions occur between sectors that are not directly adjacent anatomically but are nevertheless strongly coupled functionally, radiomically, metabolically, immunologically, or prognostically. Our aim is therefore to determine whether a relation between biomedical correlation and effective disease connectivity can be expressed in a framework where connectivity itself is inferred from constrained accessibility. The graph *G* thus serves as the basic substrate on which all later disease-state geometry and bridge interpretation are built.

### 2.2. Emergent Biomedical Accessibility Distance

In the present framework, biomedical geometry is not postulated but derived from constrained accessibility. Given two nodes *i*,*j* ∈ *V*, the emergent accessibility distance is defined by the minimal accumulated constraint cost along admissible paths:

(3) $$d\left(i,j\right)=\underset{p:i\to j}{\inf}\sum_{e\epsilon p}C(e)$$

In the present proof-of-concept implementation, this effective accessibility distance is a shortest-path cost distance. It is not an electrical resistance distance. The mapping from edge costs to effective distance is therefore

$$d_{eff}\left(a,b;G\right)=\underset{p:a\to b}{\min}\sum_{e\epsilon p}C(e)$$

where the minimum is taken over all admissible paths connecting node *a* to node *b*. Equivalently, the functional mapping used in the numerical simulations is

$$d_{eff}=F\left(\left\{C(e)\right\}\right)=\underset{p}{\min}\sum_{e\epsilon p}C(e)$$

In applications where edge weights $w_{ij}$ are already defined as costs, one may set

$$C_{ij}=w_{ij}$$

If edge weights represent affinity, similarity, or dependence strength, they should first be transformed into costs using a monotone decreasing transformation, for example:

$$C_{ij}=\frac{1}{w_{ij}+\varepsilon }$$

with *ε* > 0 used to avoid division by zero. In the representative simulations of the present study, edge costs C(e) are specified directly, and effective accessibility distances are computed using shortest-path cost distances.

If no admissible path exists from *i* to *j*, one sets *d*(*i*,*j*) = +∞. This distance does not necessarily measure anatomical separation. Rather, it measures the minimal relational cost required for accessibility between two biomedical entities. Two nodes are therefore effectively close when they are linked by a sufficiently low-cost chain of admissible biomedical relations, even if they are spatially distant, belong to different data modalities, or represent different biological scales.

This distinction is important for medical interpretation. A tumor imaging phenotype and a molecular biomarker may be anatomically or conceptually distinct, but they may be close in accessibility space if they are strongly linked through recurrent statistical, biological, or prognostic relations. Similarly, a respiratory variable and a neurological outcome may become effectively close in an ICU disease-state graph if changes in one domain systematically constrain or predict changes in the other. The accessibility distance therefore provides a general way to represent hidden biomedical proximity.

In the most general case, the induced structure is an extended quasi-metric rather than an ordinary metric, since the graph may be directed and the resulting accessibility may be asymmetric. This is medically relevant because many biological and clinical processes are direction-dependent. For example, hypoxia may influence neurological deterioration differently from the way neurological status influences oxygenation parameters. When a symmetric notion is desired, one may use the symmetrized accessibility distance

(4) $$d_{s}\left(i,j\right)=\frac{1}{2}\left(d\left(i,j\right)+d(j,i)\right)$$

provided that both directed distances are finite. For the present paper, the distinction between directed and symmetrized accessibility will be kept explicit when necessary, but most of the proof-of-concept construction may be developed using either an undirected biomedical graph or a symmetric coarse-grained accessibility observable.

This distance is the fundamental accessibility-side observable of the present study. A biomedical accessibility bridge will not mean a physical tunnel, a guaranteed causal route, or a directly observed anatomical channel. Rather, it will mean an anomalously low accessibility separation between two otherwise distant biomedical sectors. In this way, hidden disease connectivity is defined operationally before any mechanistic interpretation is imposed.

### 2.3. Biomedical Relational Dynamics and Statistical Description

A single biomedical relational configuration is not yet sufficient to support macroscopic interpretation. Medical systems are heterogeneous, noisy, patient-dependent, and often only partially observed. As in the foundational framework, we therefore consider an ensemble of admissible relational configurations and a statistical weighting over them. Let Ω denote the set of admissible configurations $G=\left(V,E,C\right)$. To each configuration, we assign an informational action *S*(*G*), interpreted here as a measure of global biomedical constraint tension. In its minimal additive form,

(5) $$S\left(G\right)=\sum_{e\epsilon E}\Phi (C\left(e\right))$$

where Φ is a monotone increasing function of the constraint strength. The precise choice of Φ is not fixed at this stage. What is required is only that a higher constraint cost corresponds to greater relational tension and that the resulting ensemble is well defined.

The corresponding Gibbs-type distribution over biomedical relational configurations is then

(6) $$P\left(G|\theta \right)=\frac{1}{Z(\theta )}e^{-\beta S(G;\theta )}$$

where *β* ≥ 0 is an inverse-noise parameter, *θ* denotes any set of coarse control parameters, and *Z*(*θ*) is the normalization factor. In biomedical terms, *θ* may include disease stage, treatment condition, imaging modality, patient subgroup, biological context, or model hyperparameters. The parameter *β* controls how strongly high-tension configurations are suppressed relative to lower-cost configurations.

At this level, macroscopic observables are expectation values over an ensemble rather than properties of a single graph. This statistical description is indispensable for the present work, since the correlation side of the proposed model will be expressed in terms of non-factorizable dependencies between coarse-grained biomedical sectors. Such dependencies are naturally defined relative to a probabilistic description over relational states.

Moreover, the same statistical structure allows one to describe how changes in graph constraints may shift the probability of coarse-grained disease configurations. In clinical language, such shifts may correspond to changes in progression risk, recurrence tendency, deterioration, treatment response, or stabilization. Here, the ensemble provides the setting in which sector-level biomedical correlation and bridge-like accessibility compression can be treated as coupled coarse-grained phenomena.

### 2.4. Coarse-Graining and Effective Biomedical Sectors

The notion of coarse-graining is central to the present biomedical extension and must be defined explicitly. By coarse-graining, we mean the replacement of detailed low-level biomedical structure by collective observables or effective sectors whose behavior is summarized by macroscopic descriptors rather than by the full graph data. In other words, fine-grained configurations distinguish every node, edge, and local constraint, whereas coarse-grained descriptions group many biomedical variables into larger subsystems characterized by aggregate accessibility and correlation properties.

Let *A*,*B* ⊆ *V* denote two such coarse-grained biomedical sectors. These sectors may be defined in several ways depending on the medical application. In radiomics, they may represent enhancing tumor, necrotic core, edema, peritumoral tissue, organ-at-risk subregions, or imaging feature modules. In oncology, they may represent primary tumor, metastatic niche, immune microenvironment, vascular interface, or treatment-response domains. In critical care, they may represent respiratory, metabolic, neurological, inflammatory, or hemodynamic sectors. In systems medicine, they may correspond to biomarker clusters, molecular pathways, organ systems, or disease-state modules.

The present construction does not depend on a unique biomedical coarse-graining prescription. What matters is that A and B represent subsystems large enough to admit collective observables and small enough to remain distinguishable within the ensemble description.

For two coarse-grained biomedical sectors, A and B, a natural effective accessibility observable is the mean inter-sector distance

(7) $$D\left(A,B\right)=\frac{1}{\left|A\right|\left|B\right|}\sum_{a\epsilon A}\sum_{b\epsilon B}d(a,b)$$

or, when needed, the corresponding symmetrized version built from *d*_s_. The quantity *D*(*A*,*B*) provides a macroscopic measure of effective separation between biomedical sectors. It is this inter-sector accessibility, rather than microscopic node-to-node distance alone, that will play the role of effective disease-state connectivity in the present theory.

Coarse-graining also applies to biomedical states. Let *x_A_* and *x_B_* denote coarse observables associated with sectors A and B, respectively. These may represent, for example, radiomic texture summaries, mean internal accessibility, local constraint statistics, biomarker activity, physiological instability, bridge occupancy, motif content, or reduced descriptors extracted from the full configuration G. The ensemble distribution over graphs then induces joint and marginal distributions over such sector variables, thereby making it possible to define non-factorizable biomedical correlations between them. Thus, the passage from detailed configurations G to coarse observables (*x_A_*, *x_B_*) is the precise step through which hidden inter-sector coupling becomes formulable.

### 2.5. Stable Biomedical Motifs as Intrinsic Sector Carriers

The foundational framework identifies stable relational motifs as connected subgraphs that remain localized, persistent, and identifiable under coarse-graining. In the biomedical setting, such motifs may be interpreted as recurrent disease-relevant patterns: stable radiomic signatures, persistent biomarker modules, characteristic physiological states, tumor microenvironment configurations, treatment-response patterns, or multivariable clinical phenotypes.

These motifs provide a more intrinsic candidate for sector definition than arbitrary graph partitions. For example, in neuro-oncology, a motif may correspond to a recurrent combination of enhancing tumor volume, necrotic fraction, edema burden, and texture heterogeneity. In critical care, a motif may correspond to a coupled pattern of hypoxemia, acidosis, lactate elevation, and neurological impairment. In recurrence modeling, a motif may correspond to a stable configuration of risk factors that persists across patients or time points.

For this reason, the sectors A and B used in the present work may be interpreted in two complementary ways. At the more general level, they are arbitrary coarse-grained biomedical subsystems. At the more intrinsic level, they may be taken to be neighborhoods supporting stable biomedical motifs *H_α_* and *H_β_*. This second interpretation is especially useful for medical applications, since it allows one to speak of a correlated pair of disease-relevant motifs whose joint organization may be encoded by an accessibility bridge.

In this view, hidden disease connectivity does not necessarily occur between isolated variables but between organized biomedical motifs. A primary tumor motif and a metastatic niche motif, a tumor-core motif and an edema motif, or a respiratory-instability motif and a neurological-outcome motif may become effectively linked if their correlation is strong enough to reduce their accessibility distance in the disease-state graph.

### 2.6. Scope of the Present Biomedical Construction

The structures introduced above are sufficient for the present paper, but their scope should be stated carefully. We are not yet working with a validated mechanistic model of tumor invasion, metastatic dissemination, organ failure, radiotherapy response, or ICU deterioration. The metric structure presently available is an accessibility geometry derived from relational constraints, and the statistical structure is a Gibbs-type ensemble over biomedical graph configurations. Within this domain, one can meaningfully formulate a correspondence between strong non-factorizable biomedical correlation and anomalously low effective accessibility separation.

This correspondence should be interpreted as structural and hypothesis-generating, not as proof of direct causality. A low-cost accessibility bridge may suggest a hidden route of disease coupling, but its biological meaning must be validated using independent clinical, imaging, molecular, physiological, or experimental evidence. The present model, therefore, provides a mathematical language for detecting and quantifying candidate hidden connectivity, rather than a complete mechanistic account by itself.

### 2.7. Notation Used in the Present Paper

For clarity and to support the transition to the correlation and bridge observables introduced in the following sections, the main symbols used throughout the paper are summarized in Table 3.

These definitions place us in a position to formulate the correlation side of the proposed framework. In the next section, we introduce a mutual-information-type measure of non-factorizable biomedical correlation between coarse-grained sectors and discuss the conditions under which such correlation may be interpreted as the informational counterpart of an emergent accessibility bridge.

## 3. Non-Factorizable Biomedical Correlation Measures

The correlation-induced accessibility-bridge model requires two conceptually distinct ingredients: a measure of biomedical coupling strong enough to identify meaningful inter-sector dependence and a notion of emergent connectivity capable of representing hidden disease bridges. Within the present framework, the second ingredient will be expressed through low-cost accessibility bridges in the emergent disease-state geometry. The first ingredient must therefore be formulated in a manner consistent with the statistical layer of the biomedical relational substrate.

Since the present theory is defined as an ensemble over admissible biomedical relational configurations rather than as a fully mechanistic causal model, the appropriate starting point is not direct biological causation but a measure of robust, non-factorizable correlation between coarse-grained biomedical sectors. The aim of this section is to define such a measure and clarify its interpretation and scope. In the present paper, this correlation is not treated as proof of causality. Rather, it is interpreted as a structured dependency that may identify candidate routes of hidden disease connectivity.

### 3.1. Coarse-Grained Biomedical Sector States

Let *A*,*B* ⊆ *V* be two coarse-grained biomedical sectors as introduced in Section 2. For each sector, we associate a reduced state variable summarizing its effective biomedical organization. Denote these by *x_A_* and *x_B_*, respectively. These variables are not assumed to be unique or universal. Rather, they represent a chosen set of coarse observables extracted from the full biomedical relational configuration G.

Depending on the medical application, such observables may include radiomic texture summaries, tumor-volume descriptors, edema or necrosis measures, local accessibility statistics, biomarker activity, physiological instability indices, treatment-response indicators, bridge occupancy, mean constraint density, or any other reduced descriptors that remain stable under the chosen coarse-graining procedure. In a neuro-oncology application, for example, *x_A_* may describe an enhancing tumor sector, while *x_B_* may describe peritumoral edema or a distant recurrence-prone region. In an ICU application, *x_A_* may represent oxygenation or arterial blood-gas status, while *x_B_* may represent neurological outcome or systemic deterioration.

Since the framework assigns a Gibbs-type probability distribution *P*(*G*|*θ*) over biomedical relational configurations, the variables *x_A_* and *x_B_* inherit an induced joint distribution

(8) $$P(x_{A},x_{B})$$

together with the corresponding marginals $P(x_{A})$ and $P(x_{B})$. This induced distribution contains the coarse-grained information needed to quantify biomedical dependence between the two sectors. If the two sectors were statistically independent, one would have

(9) $$P\left(x_{A},x_{B}\right)=P(x_{A})P(x_{B})$$

Departures from this factorized form are the natural signal of inter-sector biomedical correlation. In the present framework, however, bridge-relevant coupling will not be identified with arbitrary statistical dependence. Instead, it will be associated with sufficiently strong, structured, and persistent non-factorizability that survives coarse-graining and can be represented as a modification of effective accessibility.

### 3.2. Mutual Information as the Primary Biomedical Correlation Measure

The most natural first measure of non-factorizable biomedical coupling is the mutual information between the coarse-grained sector variables [8]:

(10) $$T\left(A:B\right)=\sum_{x_{A},x_{B}}P\left(x_{A},x_{B}\right)\log\frac{P\left(x_{A},x_{B}\right)}{P\left(x_{A}\right)P\left(x_{B}\right)}$$

When *x_A_* and *x_B_* are continuous variables, the corresponding integral form may be used. The quantity $T\left(A:B\right)$ is always nonnegative and vanishes if and only if the two sector variables are statistically independent. It therefore provides a direct measure of how strongly the two biomedical sectors fail to factorize at the coarse-grained level.

Several features make mutual information especially appropriate for the present work. First, it is naturally compatible with the statistical formulation introduced above. No assumption of direct causality, linearity, or predefined mechanistic pathway is required for its definition. Second, it quantifies total statistical dependence rather than only linear association, making it suitable for complex biomedical systems in which interactions may be nonlinear, multimodal, or distributed across several variables. Third, because the framework interprets disease-state proximity through constrained accessibility, a mutual-information-type quantity provides a direct bridge between biomedical statistics and emergent connectivity.

For these reasons, $T\left(A:B\right)$ will serve as the primary correlation-side observable throughout the present paper. The central hypothesis explored below is that sufficiently large $T\left(A:B\right)$ can induce, select, or reveal an emergent low-cost accessibility bridge between the corresponding biomedical sectors after coarse-graining.

### 3.3. Biomedical Coupling as an Operational Correlation Proxy

Although mutual information is a standard measure of total statistical dependence, the phrase “biomedical coupling” requires careful interpretation. We do not claim that the coarse-grained variables *x_A_* and *x_B_* are necessarily linked by a direct causal mechanism, nor do we claim that $T\left(A:B\right)\;$ by itself distinguishes causal influence from shared background effects, confounding, common drivers, or indirect associations. Such claims would require additional biological, clinical, imaging, temporal, interventional, or experimental validation.

More precisely, we define non-factorizable biomedical coupling as the presence of robust correlation between coarse-grained sectors or motifs that cannot be removed by the chosen reduction in microscopic detail and that remains capable of influencing or marking emergent accessibility structure. In this sense, the term is meant to capture a medically relevant dependency at the level available to the model: not necessarily a direct mechanism, but a correlation structure strong enough to act as a candidate marker of hidden disease connectivity.

This distinction is essential. The purpose of the present paper is not to replace mechanistic biomedical explanation with graph terminology but to identify the minimal informational structure required to support accessibility-bridge formation in disease-state networks. Mutual information is therefore used here as an operational proxy for structured inter-sector dependence, with the understanding that future work may refine this notion using causal inference, longitudinal data, perturbation experiments, multimodal validation, or mechanistic biological modeling.

Accordingly, throughout the remainder of the paper, the term “biomedical coupling” should be read as shorthand for a non-factorizable correlation proxy defined at the coarse-grained ensemble level. It is not used as a claim that the variables *x_A_* and *x_B_* are directly causally connected, nor that $T\left(A:B\right)$ separates causal from non-causal associations. This terminological restriction is important: the present construction targets the structural correlation–connectivity content of biomedical networks, not a complete mechanistic theory of disease propagation.

### 3.4. Bridge-Relevant Biomedical Correlation and Correlation Thresholds

Not every nonzero correlation between sectors A and B should be expected to induce or reveal a meaningful biomedical accessibility bridge. Weak, transient, noisy, confounded, or highly delocalized correlations may be too small to survive coarse-graining into an effective structural feature. For that reason, it is useful to distinguish generic biomedical dependence from bridge-relevant correlation, meaning correlation strong enough to plausibly correspond to an accessibility shortcut in disease-state space.

We therefore introduce a threshold *I_c_* > 0 and say that two sectors lie in a bridge-relevant correlation regime whenever

(11) $$T\left(A:B\right)\geq I_{c}$$

The threshold *I_c_* is not universal. Its numerical value depends on the biomedical graph ensemble, the choice of sector observables, the coarse-graining prescription, the number of patients or samples, the noise level, the imaging or clinical modality, and the scale at which disease-state geometry is probed. Conceptually, however, the role of *I_c_* is clear: it marks the point beyond which inter-sector correlation becomes large enough to be represented not merely as a statistical relation but as a candidate structural modification of effective accessibility.

In practical applications, the threshold *I_c_* should be selected relative to a null distribution rather than fixed a priori as a universal constant. For a given pair of sectors A and B, one may generate a null distribution of $T\left(A:B\right)$ by randomly permuting sample labels, sector assignments, or graph-sector memberships while preserving the marginal structure of the data. The bridge-relevant threshold can then be chosen as a high percentile of this null distribution, for example

$$I_{c}\left(A:B\right)=Q_{0.95}[T_{null}\left(A:B\right)]$$

where $I_{c}\left(A:B\right)$ is the pair-specific correlation threshold for sectors A and B, $Q_{0.95}$ denotes the 95th percentile of the null distribution, and $T_{null}\left(A:B\right)$ denotes the values of the inter-sector dependence obtained under permutation or randomization.

Equivalently, one may require the observed value of $T\left(A:B\right)$ to exceed the null-calibrated threshold with statistical significance after correction for multiple tested sector pairs. When many pairs of biomedical sectors are evaluated, false-discovery-rate control should be applied to reduce the probability of identifying spurious bridge-relevant correlations.

Uncertainty in $I_{c}$ and in the observed value of $T\left(A:B\right)$ may also be assessed by bootstrap resampling. In this case, a sector pair is considered bridge-relevant only if the bootstrap confidence interval of $T\left(A:B\right)$ remains consistently above the null-calibrated threshold. Thus, in empirical biomedical datasets, $I_{c}$ should be interpreted as a permutation- and uncertainty-calibrated operational threshold, not as a fixed biological constant.

In the proof-of-concept simulations presented in this work, $I_{c}$ is not used as a clinically optimized cutoff. Instead, the correlation proxy is swept over a predefined range, and the resulting accessibility compression is evaluated continuously. This allows the simulations to test whether increasing sector-level dependence produces systematic bridge-like compression without assuming a disease-specific clinical threshold.

One may then define a set of strongly coupled biomedical sector pairs,

(12) $${ℇ}_{I_{c}}=\left\{\left(A,B\right):T\left(A:B\right)\geq I_{c}\right\}$$

which may be regarded as the candidate bridge-relevant set of sector pairs in the ensemble. The main task of the next section will be to determine when elements of ${ℇ}_{I_{c}}$ admit a corresponding accessibility-bridge representation in the emergent biomedical geometry.

### 3.5. Conditional and Background-Subtracted Biomedical Correlation

In many biomedical settings, some portion of the observed correlation between A and B may be attributable to shared background factors rather than to a more specific pairwise relation. Such factors may include disease stage, age, treatment type, scanner protocol, tumor burden, global inflammation, systemic severity, comorbidity load, cohort composition, or overall network density. If one wishes to isolate the part of the dependence most likely to contribute to bridge formation, it is useful to define a conditional or background-subtracted measure of correlation.

Let *Y* denote a collection of global or environmental biomedical observables, such as disease stage, total tumor volume, treatment group, global inflammatory state, scanner/site variable, global constraint density, overall connectivity class, or patient subgroup. Then one may define conditional mutual information

(13) $$T\left(A:B|\Upsilon \right)=\sum_{x_{A},x_{B},\gamma }P\left(x_{A},x_{B},\gamma \right)\log\frac{P\left(x_{A},x_{B}|\gamma \right)}{P\left(x_{A}|\gamma \right)P\left(x_{B}|\gamma \right)}$$

This quantity measures the residual dependence between A and B after controlling for the background variable *Y*. In numerical and clinical studies, $T\left(A:B|\Upsilon \right)\;$ may prove useful for separating genuinely pair-specific biomedical dependence from more diffuse cohort-wide or disease-stage effects.

For the conceptual development of the present paper, the unconditioned quantity $T\left(A:B\right)$ will be sufficient. Nonetheless, the conditional form shows that the framework can be refined systematically when stronger discrimination between direct, indirect, confounded, and background-mediated correlation becomes necessary.

### 3.6. Practical Estimation of Biomedical Mutual Information

Although the present paper uses $T\left(A:B\right)$ as a theoretical measure of non-factorizable biomedical coupling, its estimation in real biomedical datasets requires practical choices depending on the type, dimensionality, and sample size of the available variables. For discrete or discretized sector variables, $T\left(A:B\right)$ may be estimated using empirical joint and marginal frequencies, preferably with bias correction or permutation-based calibration when sample sizes are limited. For continuous variables, nonparametric estimators such as k-nearest-neighbor mutual information estimators may be used to avoid imposing a linear dependence structure [25,26].

In small biomedical cohorts, estimated mutual information values should not be interpreted without uncertainty assessment. Permutation testing can be used to test whether the observed dependence exceeds the level expected under random sector-label reassignment or sample shuffling. Bootstrap resampling can provide confidence intervals for $T\left(A:B\right)$, bridge-relevant thresholds, and downstream accessibility-compression observables. When many sector pairs or motif pairs are tested, false-discovery-rate correction should be applied to reduce the risk of identifying spurious bridge-relevant correlations [27].

In addition, biomedical dependence may be influenced by shared background variables such as disease stage, scanner protocol, treatment group, age, global tumor burden, systemic inflammation, or cohort composition. In such settings, the conditional mutual information $T\left(A:\left.B\right|\Upsilon \right)$ introduced above provides a natural extension for estimating residual inter-sector coupling after accounting for relevant background factors. Thus, in practical applications, bridge-relevant biomedical coupling should be interpreted as a robust, uncertainty-aware, and, when possible, background-adjusted mutual information estimate rather than as a raw correlation value.

### 3.7. Motif-Based Biomedical Coupling

As emphasized in the foundational framework, stable relational motifs provide intrinsic carriers of persistent organization. In the biomedical setting, such motifs may correspond to stable disease-relevant subgraphs: radiomic signatures, multivariable clinical phenotypes, tumor microenvironment patterns, biomarker modules, physiological deterioration profiles, recurrence-risk signatures, or treatment-response configurations. These motifs are persistent, identifiable under coarse-graining, and potentially comparable across patients, time points, or disease subtypes.

Let *H_α_* and *H_β_* be two stable biomedical motifs, together with the coarse-grained neighborhoods supporting them. The induced coarse observables associated with these neighborhoods define a mutual information

(14) $$T\left(H_{\alpha }:H_{\beta }\right)$$

which may be interpreted as a motif-level biomedical coupling measure. A pair of motifs satisfying

(15) $$T\left(H_{\alpha }:H_{\beta }\right)\geq I_{c}$$

will be regarded as a candidate bridge-relevant motif pair.

This formulation is more intrinsic than one based on arbitrary graph partitions, because it connects the correlation side directly to recurrent biomedical structures. For example, one may ask whether a tumor-core radiomic motif and an edema-related motif are strongly coupled; whether a hypoxia-related physiological motif and a poor neurological outcome motif show persistent dependence; or whether a primary tumor motif and a metastatic niche motif become effectively linked in the disease-state graph.

This motif-based interpretation will be particularly useful later, when accessibility bridges are discussed not merely as shortcuts between arbitrary sectors but as structural encodings of strongly coupled disease-relevant motifs.

### 3.8. Correlation-Induced Biomedical Accessibility Hypothesis

The definitions above motivate the central working hypothesis of the present paper:

Correlation-Induced Biomedical Accessibility Hypothesis. There exists a regime of coarse-grained biomedical organization in which a sufficiently strong non-factorizable correlation between two sectors A and B is encoded, after coarse-graining, by an anomalous reduction in their effective accessibility distance.

In symbolic form, this means that for some monotone decreasing function *f*,

(16) $$D_{eff}\left(A,B\right)=f\left(T\left(A:B\right)\right),\;\;\;\;f^{\prime }(T)<0$$

where $D_{eff}\left(A,B\right)$ denotes the effective inter-sector accessibility distance introduced in Section 2. At the present stage, this relation is an ansatz to be motivated and tested, not a theorem. Its significance is that it states precisely what a correlation-induced accessibility-bridge model means in the biomedical relational framework: strong sector-level correlation becomes associated with effective disease-state compression.

The next section develops this idea geometrically by introducing bridge observables and defining the conditions under which reduced accessibility distance may be interpreted as a hidden biomedical accessibility bridge.

### 3.9. Summary of Correlation-Side Observables

This section has defined the correlation-side observables used throughout the paper. Coarse-grained biomedical sector variables x_A_ and x_B_ are induced from the ensemble over relational configurations, and their non-factorizable dependence is quantified primarily by the mutual-information-type measure $T\left(A:B\right)$. In biomedical applications, this quantity should be estimated with appropriate attention to estimator choice, uncertainty assessment, background adjustment, and multiple-testing control. Within the present framework, biomedical coupling is therefore interpreted structurally as robust, coarse-grained, non-factorizable correlation rather than as direct causality. Bridge-relevant correlation is identified by the threshold condition $T\left(A:B\right)\geq I_{c}$, with stable biomedical motifs providing an intrinsic realization of such sector pairs. With these definitions in place, we may now turn to the bridge side of the framework and construct explicit geometric observables for hidden biomedical accessibility-bridge formation.

## 4. Emergent Biomedical Bridge Geometry and Accessibility-Side Observables

Having defined the correlation side of the framework in terms of robust, non-factorizable biomedical dependence between coarse-grained sectors, we now turn to the accessibility side. In the present framework, the bridge side of the model is not expressed in terms of physical anatomical tunnels, predefined causal routes, or fixed mechanistic pathways. Instead, it is formulated directly in the language of emergent biomedical accessibility geometry. The task of this section is therefore to define hidden disease connectivity in a way native to the biomedical relational substrate: not as a necessarily anatomical channel but as an anomalously low-cost bridge in the accessibility structure linking two otherwise distant coarse-grained biomedical sectors.

The conceptual point is straightforward. Since effective disease-state geometry is derived from minimal relational path cost, a biomedical bridge should correspond to a configuration in which the accessibility separation between two sectors is strongly reduced relative to the ambient organization of the graph. Such a reduction must be structural rather than incidental: it should persist under coarse-graining, be attributable to a well-defined bridge-like subset of relations, and be measurable through observables that distinguish bridge-induced compression from ordinary background connectivity. These requirements motivate the definitions introduced below.

### 4.1. Effective Inter-Sector Biomedical Accessibility

Let *A*,*B* ⊆ *V* be two coarse-grained biomedical sectors. As introduced in Section 2, a natural macroscopic measure of their separation is the mean inter-sector accessibility distance

(17) $$D\left(A,B\right)=\frac{1}{\left|A\right|\left|B\right|}\sum_{a\epsilon A}\sum_{b\epsilon B}d(a,b)$$

where *d*(*a*,*b*) is the emergent accessibility distance on the underlying biomedical relational graph. If the graph is directed and asymmetry is relevant, one may instead work with the ordered distance *D*(*A* → *B*) or with the symmetrized form built from *d_s_*(*a*,*b*). For the present development, the precise choice is secondary; what matters is that *D*(*A*,*B*) quantifies the coarse-grained accessibility separation between the two biomedical sectors in the emergent disease-state geometry.

This quantity will serve as the primary accessibility-side distance observable. A hidden biomedical bridge will correspond to a regime in which *D*(*A*,*B*) becomes anomalously small relative to the background large-scale structure of the graph. In clinical terms, two sectors may become effectively close even if they are anatomically distant, belong to different modalities, or represent different biological scales, provided that the relational cost of accessibility between them is sufficiently reduced.

### 4.2. Candidate Biomedical Bridge Sets

To identify a bridge-like structure more explicitly, let

(18) $$W_{AB}\subseteq E$$

denote a distinguished subset of admissible biomedical relations that potentially mediates accessibility between sectors A and B. The set $W_{AB}$ may be interpreted as a candidate biomedical bridge set if it satisfies two qualitative conditions:

1. its removal significantly increases the effective accessibility distance between A and B;
2. its presence contributes a disproportionately large share of the low-cost paths connecting the two sectors.

In general, $W_{AB}$ need not consist only of direct edges from A to B. It may also include a connected relay structure of intermediate nodes and edges whose net effect is to provide an accessibility shortcut between the two sectors. In biomedical settings, such relay structures may correspond to intermediate imaging features, biomarker modules, physiological variables, tissue compartments, microenvironmental factors, or clinical-state transitions. Thus, a bridge is defined operationally through its role in accessibility compression rather than through any fixed graph-theoretic or anatomical shape.

Let ${G\backslash W}_{AB}$ denote the graph obtained by deleting the bridge candidate set. The corresponding background accessibility distance is then

(19) $$D_{bg}\left(A,B;W_{AB}\right)=\frac{1}{\left|A\right|\left|B\right|}\sum_{a\epsilon A}\sum_{b\epsilon B}d_{{G\backslash W}_{AB}}(a,b)$$

whenever the distances remain finite. If deletion disconnects the sectors, the corresponding terms may be treated as +∞, signaling that the bridge candidate is structurally essential. This comparison immediately provides a way to quantify the accessibility impact of the bridge.

### 4.3. Biomedical Bridge Gain and Accessibility Compression

The simplest measure of bridge effectiveness is the ratio between background and actual inter-sector separation. We therefore define the biomedical bridge gain

(20) $$\Gamma \left(A,B;W_{AB}\right)=\frac{D_{bg}\left(A,B;W_{AB}\right)}{D(A,B)}$$

By construction, Γ ≥ 1 whenever the candidate set $W_{AB}$ reduces the effective separation between sectors. Large values of Γ indicate strong accessibility compression and therefore strong bridge behavior. In particular, if the removal of $W_{AB}$ disconnects the two sectors entirely, then $D_{bg}\left(A,B;W_{AB}\right)=+\infty$, and the bridge gain becomes formally unbounded. This corresponds to the strongest possible bridge regime: the sectors remain effectively connected only because the bridge set exists.

Equivalently, one may define a normalized accessibility compression

(21) $${∆}_{B}\left(A,B;W_{AB}\right)=1-\frac{D(A,B)}{D_{bg}\left(A,B;W_{AB}\right)}$$

for finite *D*_bg_. The quantity Δ_B_ lies in [0,1) and measures the fractional reduction in effective separation caused by the bridge set. Both Γ and Δ_B_ will be useful in numerical work. The former highlights multiplicative gain; the latter emphasizes relative compression.

These observables provide the first precise sense in which a biomedical accessibility bridge can be defined within the present framework: not by physical topology, anatomical continuity, or assumed causality, but by measurable reduction in coarse-grained accessibility distance.

### 4.4. Biomedical Accessibility-Bridge Criterion

We now define the central accessibility-side notion of the paper.

A candidate bridge set $W_{AB}$ will be said to realize a biomedical accessibility bridge between sectors A and B if the following conditions hold:

1. **Accessibility compression:**

(22) $$\Gamma \left(A,B;W_{AB}\right)\geq \Gamma_{c}$$

for some prescribed threshold Γ_c_ > 1;

2. Structural localization: the reduction in distance is attributable primarily to a bounded subset $W_{AB}$ rather than to diffuse changes across the entire graph;
3. Coarse-grained persistence: the induced reduction in *D*(*A*,*B*) survives under the chosen coarse-graining and is visible at the level of sector observables;
4. Stability: the bridge-supporting configuration is not an isolated combinatorial accident but remains statistically favored or dynamically persistent under admissible perturbations, resampling, or model variation.

The threshold Γ_c_ is ensemble-dependent and need not be universal. It marks the point at which accessibility compression becomes large enough to justify interpreting the bridge as a meaningful biomedical structure rather than as a weak background fluctuation.

In practical applications, $\Gamma_{c}$ should be selected relative to a matched graph-null model rather than fixed as a universal constant. For a given sector pair A and B and a candidate bridge set $W_{AB}$, one may generate null graphs by degree-preserving rewiring, weight-preserving rewiring, random non-bridge edge modulation, or generic inter-sector modulation that does not specifically target the candidate bridge set. For each null graph, the corresponding bridge gain is computed, producing a null distribution of bridge gain values.

The critical accessibility gain threshold may then be chosen as a high percentile of this null distribution, for example

$$\Gamma_{c}\left(A,B;W_{AB}\right)=Q_{0.95}\left[\Gamma_{null}\left(A,B;W_{AB}\right)\right]$$

where $\Gamma_{c}\left(A,B;W_{AB}\right)$ is the pair- and bridge-specific critical gain threshold, $Q_{0.95}$ denotes the 95th percentile of the null bridge gain distribution, and $\Gamma_{null}\left(A,B;W_{AB}\right)$ denotes the bridge gain values obtained under the matched graph-null model.

Equivalently, a candidate bridge may be accepted only if its observed gain $\Gamma \left(A,B;W_{AB}\right)$ exceeds the null-calibrated threshold and remains stable under resampling or model perturbation. When many sector pairs or bridge candidates are tested, multiple-testing correction should be applied to reduce the risk of identifying spurious accessibility bridges.

In clinical applications, $\Gamma_{c}$ may also be constrained by a predefined minimal meaningful accessibility compression, but such a cutoff should be selected only within a training or calibration procedure. Independent external validation would be required before assigning diagnostic, prognostic, or mechanistic clinical meaning to any bridge defined by $\Gamma \left(A,B;W_{AB}\right)\geq \Gamma_{c}$.

In this way, a biomedical bridge is defined operationally and structurally. It is simply a localized, stable, high-gain shortcut in the emergent disease-state accessibility geometry.

### 4.5. Parametric Biomedical Bridge Families

To study the opening and closing of bridges, it is useful to introduce a control parameter λ that modulates the cost of edges belonging to the bridge candidate set $W_{AB}$. Let

(23) $$C_{\lambda }\left(e\right)=\left\{\begin{array}{c} \lambda C_{0}\left(e\right),\;e\in W_{AB}\\ C_{0}\left(e\right),\;e\notin W_{AB} \end{array}\right.\;\;\;\;\;\;\;\;\;\lambda >0$$

where $C_{0}\left(e\right)$ is the unperturbed constraint structure. When 0 < λ < 1, the bridge edges become less costly and the bridge effectively opens; when λ > 1, the bridge becomes more strongly constrained and tends toward closure. The associated λ-dependent accessibility distance is

(24) $$d_{\lambda }\left(i,j\right)=\underset{p:i\to j}{\inf}\sum_{e\epsilon p}C_{\lambda }(e)$$

with corresponding sector distance

(25) $$D_{\lambda }\left(A,B\right)=\frac{1}{\left|A\right|\left|B\right|}\sum_{a\epsilon A}\sum_{b\epsilon B}d_{\lambda }(a,b)$$

In biomedical terms, the parameter λ can be interpreted as a generic modulation of bridge accessibility. Depending on the application, such modulation may represent increased or decreased biological compatibility, treatment effect, inflammatory activation, vascular permeability, metabolic coupling, radiomic similarity, physiological instability, or statistical transition probability.

**Monotonicity of Accessibility Under Bridge Opening**

**Proposition 1.**  *Let* $G=\left(V,E,C_{0}\right)$ *be a weighted biomedical relational graph with strictly positive edge costs, and let W_AB_ ⊆ E be a candidate bridge set. Define the bridge-modulated cost function*

$$C_{\lambda }\left(e\right)=\left\{\begin{array}{c} \lambda C_{0}\left(e\right),\;\;\;\;\;\;\;\;\;e\in W_{AB}\\ C_{0}\left(e\right),\;\;\;\;\;\;\;\;\;\;\;\;e\notin W_{AB} \end{array}\right.\lambda >0$$

*If 0 < λ*_1_ *≤ λ*_2_*, then for all i,j ∈ V,*

$$d_{\lambda_{1}}(i,j)\leq d_{\lambda_{2}}(i,j)$$

*and therefore*

$$D_{\lambda_{1}}(A,B)\leq D_{\lambda_{2}}(A,B)$$

**Proof.**  For any admissible path *p*:*i*→*j*, the path cost under *C*_λ_ is

$$L_{\lambda }\left(p\right)=\sum_{e\epsilon p}C_{\lambda }(e)$$

If 0 < λ_1_ ≤ λ_2_, then $C_{\lambda_{1}}(e)\leq C_{\lambda_{2}}(e)$ for every e ∈ *W_AB_*, while all non-bridge costs are unchanged. Hence

$$L_{\lambda_{1}}\left(p\right)\leq L_{\lambda_{2}}\left(p\right)$$

for every admissible path *p*. Taking the infimum over all paths from *i* to *j* gives

$$d_{\lambda_{1}}(i,j)\leq d_{\lambda_{2}}(i,j)$$

Averaging this inequality over all *a* ∈*A* and *b* ∈*B* yields

$$D_{\lambda_{1}}(A,B)\leq D_{\lambda_{2}}(A,B)$$

Thus, reducing the cost of a candidate bridge set cannot increase the effective inter-sector accessibility distance. □

**Interpretation.**

This proposition does not by itself prove a biomedical mechanism or establish causality. Rather, it establishes the mathematical consistency of the bridge-side mechanism: once a candidate bridge set is selected, lowering its relational cost produces monotonic accessibility compression. The nontrivial question addressed by the coupling model and simulations is whether a strong biomedical correlation selects, stabilizes, or reveals precisely such bridge-cost reductions.

This one-parameter family provides a natural way to study bridge modulation. In the present framework, one does not speak of a physical opening or anatomical passage. Instead, one studies the deformation of accessibility compression as the bridge cost is varied. A stronger biomedical bridge corresponds to smaller $D_{\lambda }\left(A,B\right)$, larger Γ_λ_, and higher effective connectivity between the sectors.

### 4.6. Biomedical Bridge Tension and Effective Opening Response

The statistical layer of the framework allows one to assign an effective response to changes in bridge cost. Let λ be treated as a coarse control parameter entering the informational action. The free energy is then

(26) $$F\left(\lambda \right)=-\beta^{-1}\log Z(\lambda )$$

where

(27) $$Z\left(\lambda \right)=\sum_{G\in \Omega }e^{-\beta S(G;\lambda )}$$

Following the ensemble-response formalism introduced above, we define the effective bridge response

(28) $$R_{\lambda }=-\frac{\partial F}{\partial \lambda }$$

This quantity measures the system-level tendency to open or close the bridge under constraint modulation. If $R_{\lambda }<0$, decreasing λ lowers the free energy, so the system favors bridge opening. If $R_{\lambda }>0$, a stronger bridge cost is favored, and the accessibility shortcut tends to collapse.

In biomedical terms, $R_{\lambda }$ may be interpreted as an ensemble-level tendency toward bridge stabilization or suppression. For example, a disease configuration may statistically favor increased coupling between two sectors under certain pathological conditions, whereas treatment or stabilization may increase the effective bridge cost and weaken the shortcut. This interpretation remains structural and model-based; it does not imply a direct biological mechanism unless supported by additional evidence.

The corresponding biomedical question is, therefore, under what relational and statistical conditions is a low-cost accessibility bridge dynamically or statistically stabilized?

### 4.7. Curvature Signatures of Biomedical Bridge Regions

The foundational framework also defines curvature-like observables through transport-based accessibility structure, including Ollivier–Ricci curvature. These observables can be used to characterize bridge regions more geometrically. Let *κ*(*x,y*) denote the curvature assigned to an edge or local biomedical relation (*x,y*). Then, for a candidate bridge set $W_{AB}$, define the mean bridge curvature

(29) $$\kappa_{W_{AB}}=\frac{1}{\left|W_{AB}\right|}\sum_{\left(x,y\right)\in W_{AB}}\kappa (x,y)$$

Similarly, let *κ*_ext_ denote the mean curvature in a surrounding reference region not belonging to the bridge. The contrast

(30) $${∆\kappa }_{AB}=\kappa_{W_{AB}}-\kappa_{ext}$$

provides a geometric signature of the bridge region relative to the ambient accessibility structure.

Although the present paper does not require a full curvature analysis to define accessibility-bridge observables, such quantities may prove useful in numerical and clinical studies. In particular, they offer a way to test whether high-gain accessibility shortcuts are accompanied by distinctive local distortions in the emergent disease-state geometry. This could help distinguish genuine bridge-like structures from purely combinatorial or noisy graph effects.

### 4.8. Motif-Level Biomedical Bridge Structures

As discussed previously, the most intrinsic version of the framework is obtained when the sectors A and B are not arbitrary partitions but neighborhoods supporting stable biomedical motifs *H_α_* and *H_β_*. In that setting, a biomedical accessibility bridge is a low-cost shortcut connecting the effective neighborhoods of two motif carriers. One may then write

(31) $$\Gamma \left(H_{\alpha },H_{\beta };W_{AB}\right)=\frac{D_{bg}\left(H_{\alpha },H_{\beta };W_{AB}\right)}{D\left(H_{\alpha },H_{\beta }\right)}$$

where the numerator and denominator are understood as coarse-grained distances between motif neighborhoods.

This formulation is especially useful for medical interpretation because it brings the accessibility-bridge concept into direct contact with recurrent disease-relevant patterns. Instead of asking whether two arbitrary graph regions are linked by a shortcut, one asks whether two strongly coupled biomedical motifs are encoded by an accessibility bridge. Examples include a tumor-core motif and an edema motif, a primary tumor motif and a metastatic niche motif, an inflammatory motif and a treatment-resistance motif, or a respiratory-instability motif and a neurological-outcome motif.

This is the setting in which the proposed framework becomes most clinically meaningful: hidden connectivity is interpreted at the level of stable biomedical patterns rather than isolated variables.

### 4.9. Accessibility-Side Biomedical Bridge Hypothesis

We may now state the accessibility-side hypothesis in explicit form:

Biomedical Accessibility-Bridge Hypothesis. A hidden biomedical accessibility bridge between sectors A and B exists whenever there is a localized candidate bridge set $W_{AB}\subseteq E$ such that the coarse-grained accessibility gain Γ(*A*,*B*;*W_AB_*) exceeds a prescribed threshold and the associated accessibility compression is stable under the relevant ensemble, resampling, or dynamical perturbations.

This hypothesis does not claim the existence of a direct anatomical route, a proven causal mechanism, or a validated biological pathway. Rather, it identifies the minimal structural content of the bridge side within the present theory: a stable, localized, high-gain reduction in accessibility distance between coarse-grained biomedical sectors. This is the notion that will be coupled to non-factorizable biomedical correlation in the next section.

### 4.10. Summary of Accessibility-Side Observables

This section has defined the accessibility-side observables used throughout the paper. The effective inter-sector distance *D*(*A*,*B*) measures biomedical accessibility between coarse-grained sectors, while $W_{AB}\subseteq E$ denotes a candidate bridge set whose removal defines the background distance *D*_bg_(*A*,*B*;*W_AB_*). Bridge strength is quantified by the gain $\Gamma (A,B;W_{AB})$ and the fractional compression Δ_B_(*A*,*B*;*W_AB_*), so that a biomedical accessibility bridge is defined operationally by high gain, localization, persistence, and stability. The modulation parameter λ controls bridge opening or closure through cost variation, and the response $R_{\lambda }=-\partial F/\partial \lambda$ provides an ensemble-level measure of bridge tendency. Bridge regions may also be characterized by curvature contrast, while motif-level bridge structures provide the most intrinsic formulation of the concept. These definitions prepare the core step of the paper: coupling biomedical correlation to accessibility compression in the relational–informational graph framework.

## 5. Coupling Biomedical Correlation to Accessibility-Bridge Geometry

Section 3 and Section 4 established the two structural components required for a correlation-induced accessibility-bridge model within the biomedical relational–informational framework. On the one hand, the correlation side was formulated in terms of robust, non-factorizable dependence between coarse-grained biomedical sectors, quantified primarily through the mutual information T(A:B). On the other hand, the accessibility side was formulated in terms of localized, stable accessibility compression, quantified through bridge sets *W_AB_*, effective inter-sector distance *D*(*A*,*B*), and bridge gain Γ(*A*,*B*;*W_AB_*). What remains is to define the coupling between these two descriptions. The purpose of the present section is therefore to specify how a strong biomedical correlation can be represented as an emergent accessibility bridge in disease-state space.

The central idea is that correlation and bridge geometry should not be treated as independent structures that coincidentally appear in the same model. Rather, they may arise as complementary coarse-grained expressions of a single underlying biomedical organization. In the present framework, this means that sufficiently strong inter-sector biomedical correlation may either induce, select, stabilize, or reveal a low-cost bridge in the emergent accessibility geometry. The coupling rules introduced below are intended as minimal realizations of this principle. They do not yet constitute a mechanistic biological derivation, but they provide a precise formalization of what a correlation-induced accessibility-bridge model means in a biomedical relational setting. Their numerical implementation is described separately in Section 6.6.

### 5.1. The Biomedical Correlation–Compression Principle

The basic structural postulate of the present paper may be stated as follows:

Biomedical Correlation–Compression Principle. In an appropriate regime of coarse-grained biomedical organization, increasing non-factorizable correlation between sectors A and B leads to decreasing effective accessibility distance between them.

This principle expresses the idea that strongly coupled biomedical sectors may become effectively closer in disease-state space, even when they are not anatomically adjacent or directly linked by an obvious causal pathway. In the current framework, the relevant distance is not necessarily physical distance but accessibility distance induced by constrained relational paths. Likewise, the relevant correlation is not assumed to prove direct causality but is represented by a coarse-grained mutual-information-type measure of biomedical dependence.

Thus, the principle should be understood as a structural and hypothesis-generating correspondence, not as a complete mechanistic theorem. It proposes that a strong biomedical coupling may be encoded geometrically as accessibility compression.

Accessibility compression is used here because the framework defines biomedical proximity through constrained relational accessibility rather than through anatomical distance alone. If two sectors become strongly coupled at the coarse-grained level, the most direct geometric expression of that coupling is therefore a reduction in the effective cost of moving, communicating, transitioning, or sharing predictive information between them in the disease-state graph. This does not imply that accessibility compression is the only possible representation of biomedical coupling. Alternative formulations could be based on diffusion distance, random-walk accessibility, electrical resistance distance, graph-embedding proximity, curvature change, or learned graph representations. The present work uses shortest-path accessibility because it provides the most transparent proof-of-concept definition of localized bridge gain.

The correlation–compression relation may also fail in several biomedical situations. A high mutual-information-type value may reflect confounding, common drivers, scanner or site effects, treatment heterogeneity, cohort imbalance, global disease severity, or diffuse network-wide changes rather than a localized bridge-like structure. In such cases, the bridge interpretation should not be accepted without background adjustment, null-model comparison, uncertainty assessment, and independent clinical or biological validation.

At the most abstract level, the principle may be written as

(32) $$D_{eff}\left(A,B\right)=f\left(T\left(A:B\right)\right),\;\;\;\;\;{\;\;\;f}^{\prime }\left(T\right)<0$$

where $D_{eff}\left(A,B\right)$ is the effective accessibility separation between sectors and *f* is a monotone decreasing function. This relation says only that a larger correlation implies stronger accessibility compression. To make the model predictive, one must specify a more explicit form of *f* or embed the coupling into the action governing the biomedical relational ensemble.

### 5.2. Minimal Exponential Coupling Ansatz

The simplest explicit realization is an exponential compression law:

(33) $$D_{eff}\left(A,B\right)=D_{0}\left(A,B\right)e^{-\alpha T\left(A:B\right)},\;\;\;\;\;\alpha >0$$

where $D_{0}\left(A,B\right)$ denotes the background effective distance between biomedical sectors in the absence of correlation-induced bridge effects, and α is a coupling parameter measuring how strongly correlation is translated into accessibility compression.

This ansatz has several advantages. First, it is monotone and positive by construction. Second, it reduces smoothly to the background value $D_{0}\left(A,B\right)$ when $T\left(A:B\right)=0$. Third, it makes strong-correlation regimes immediately visible: as $T\left(A:B\right)$ increases, the effective separation shrinks exponentially, signaling the onset of bridge-like accessibility compression.

One may equivalently define the corresponding biomedical gain factor

(34) $$\Gamma_{B}\left(A,B\right)=\frac{D_{0}\left(A,B\right)}{D_{eff}\left(A,B\right)}=e^{\alpha T\left(A:B\right)}$$

In this form, the coupling states directly that the bridge gain grows exponentially with biomedical correlation.

The exponential form is not unique, nor is it claimed to be biologically fundamental. It is introduced as a minimal phenomenological coupling law satisfying three structural requirements: positivity of the effective distance, recovery of the background distance $D_{0}\left(A,B\right)$ when $T\left(A:B\right)=0$, and monotonic accessibility compression as $T\left(A:B\right)$ increases. Alternative monotone couplings, including rational, sigmoidal, piecewise-linear, or thresholded forms, could also be used depending on the biomedical graph ensemble, disease process, and desired macroscopic behavior. The numerical conclusions should therefore be interpreted at the level of monotone correlation–compression coupling, not as evidence for a unique biological law governing biomedical connectivity.

### 5.3. Threshold Formulation of the Biomedical Correspondence

In many medical applications, the emergence of a meaningful accessibility bridge may be better treated as a threshold phenomenon than as a smooth deformation. A weak correlation may have little structural consequence, whereas beyond a certain level of coupling, a bridge-like shortcut may become detectable, stable, or clinically relevant. In that case, one may formulate the correspondence in the following way:

(35) $$T\left(A:B\right)\geq I_{c}⟹\exists W_{AB}\subseteq E\;\;\;\;\;\mathrm{such}\;\mathrm{that}\;\;\;\;\;\Gamma (A,B;W_{AB})\geq \Gamma_{c}$$

Here, *I*_c_ is the threshold for bridge-relevant biomedical correlation introduced in Section 3, and Γ_c_ is the threshold for meaningful accessibility compression introduced in Section 4. This implication expresses the correspondence in direct structural terms: when biomedical correlation exceeds a critical strength, a bridge set exists whose effect on accessibility is strong enough to qualify as a hidden biomedical bridge.

The threshold form has the advantage of being operationally clear and numerically robust. In simulations or clinical datasets, one need not establish a precise functional law for all correlation values. It may be sufficient to show that once correlation exceeds a certain regime, high-gain bridge configurations appear with enhanced probability or stability. This is often the most defensible way to demonstrate the model in proof-of-concept biomedical studies.

It is important to distinguish bridge gain from correlation sensitivity. In the present framework, bridge gain is not defined as a derivative with respect to mutual information. It is defined as a ratio between the background inter-sector distance and the correlation-modulated inter-sector distance:

$$\Gamma \left(A,B;W_{AB};I\right)=\;\frac{D_{bg}(A,B;W_{AB})}{D_{I}(A,B)}\;$$

where $D_{I}(A,B)$ denotes the effective inter-sector distance after applying the coupling associated with the inter-sector dependence level $I=T\left(A:B\right)$.

A separate sensitivity observable may be introduced as

$$\chi_{AB}\left(I\right)=-\frac{\partial D_{I}(A,B)}{\partial I}$$

This quantity measures how rapidly accessibility distance decreases as inter-sector dependence increases. Thus, Γ and $\chi_{AB}$ are related but distinct observables: Γ measures accumulated compression relative to background, whereas $\chi_{AB}$ measures the local response of accessibility distance to correlation.

Under the exponential ansatz, $D_{I}\left(A,B\right)=D_{0}\left(A,B\right)\exp\left[-\alpha I\right]$ one obtains

$$\chi_{AB}\left(I\right)=\alpha D_{I}\left(A,B\right)$$

and

$$\Gamma_{B}\left(I\right)=\frac{D_{0}\left(A,B\right)}{D_{I}\left(A,B\right)}=\exp\left[\alpha I\right]$$

Therefore, a derivative relation of the form $\Gamma_{AB}\propto -\partial D_{eff}/\partial I$ is not assumed in general. Such a relation would be model-dependent and would require a specific normalization. In the present work, bridge gain is therefore treated as a finite compression ratio, while the derivative with respect to inter-sector dependence is interpreted only as an optional local sensitivity measure.

### 5.4. Action-Based Coupling of Biomedical Correlation and Bridge Cost

A more intrinsic implementation is obtained by coupling biomedical correlation directly to the informational action governing the statistical ensemble. Let *S*_0_(*G*) denote the baseline informational action of the biomedical relational graph. We then introduce an effective action

(36) $$S_{B}\left(G;A,B\right)=S_{0}\left(G\right)-\eta T\left(A:B\right)+\mu \sum_{e\in W_{AB}}\Phi (C\left(e\right))$$

where η > 0 and μ > 0 are coupling constants. The first term is the original global constraint tension. The second term rewards configurations in which the sectors A and B are strongly correlated. The third term penalizes costly bridge structures and therefore favors low-cost bridge realizations when such sets are present.

Equivalently, this third term should be read as a selection pressure against high-cost realizations of the candidate bridge, not as a claim that a bridge is created automatically or causally by correlation alone. The action-based formulation can be understood in two complementary ways. In a fully ensemble-based model, correlation-induced bridge formation may be encoded through an explicit bridge-support or bridge-occupancy term. For example, one may write the bridge-coupled action schematically as

$$S_{B}\left(G;A,B\right)=S_{0}\left(G\right)-\eta T\left(A:B\right)B_{AB}(G)+\mu \sum_{e\in W_{AB}}\Phi (C\left(e\right))$$

where $B_{AB}(G)$ is a bridge-support score or bridge-occupancy indicator. In the simplest case, $B_{AB}\left(G\right)=1$ when the graph configuration contains or stabilizes the candidate bridge architecture $W_{AB}$, and $B_{AB}\left(G\right)=0$ otherwise. More generally, $B_{AB}(G)$ may be a continuous score measuring the degree to which the graph supports a localized low-cost bridge between sectors A and B.

This expression clarifies that the correlation term does not act on the graph in isolation; it is coupled to the presence or strength of a bridge-supporting configuration. Thus, strong inter-sector dependence lowers the effective action most strongly when the graph also supports a candidate accessibility bridge. In models where the edge set itself is dynamic, additional bridge-occupancy, edge-activation, or rewiring terms may be introduced to control the appearance and disappearance of $W_{AB}$.

Strictly speaking, $T\left(A:B\right)$ is an ensemble-level correlation measure induced by the probability distribution over the coarse-grained sector variables. Therefore, Equation (36) should be read as an effective or mean-field ensemble action unless a graph-level correlation score is specified explicitly. In the representative simulations below, we do not sample the full action-based ensemble. Instead, we use the graph-level bridge-cost modulation rule as the primary implementation, while the fully self-consistent action-based sampling formulation is retained as the natural ensemble-level extension.

The associated Gibbs ensemble is

(37) $$P\left(G|A,B\right)=\frac{1}{Z_{AB}}e^{-\beta S_{B}(G;A,B)}$$

This form realizes the correlation–accessibility coupling dynamically at the statistical level. A strong biomedical correlation lowers the effective action, while low-cost bridge realizations minimize the bridge-cost penalty. As a result, configurations combining high inter-sector correlation with low-cost bridge support acquire larger statistical weight. In this way, accessibility compression is not merely imposed after the fact but emerges as the statistically favored structural encoding of the coupled sector pair.

This action-based implementation is particularly attractive because it fits naturally into the logic of biomedical systems modeling. Disease states, treatment responses, and physiological transitions may be interpreted as ensemble-level configurations constrained by biological, clinical, imaging, and statistical costs. The present extension adds non-factorizable biomedical correlation as a structural ingredient capable of biasing the ensemble toward bridge-supporting states.

### 5.5. Correlation-Dependent Biomedical Bridge Cost

A related and often numerically convenient graph-level formulation is to let the bridge cost itself depend explicitly on biomedical correlation. For example, one may define

(38) $$C_{AB}\left(e\right)=\frac{C_{0}(e)}{1+\alpha T\left(A:B\right)},\;\;\;\;\;\;\;\;\;\;\;e\in W_{AB}$$

while keeping

(39) $$C_{AB}\left(e\right)=C_{0}\left(e\right),\;\;\;\;\;\;\;\;\;\;\;\;\;e\notin W_{AB}$$

Under this rule, stronger biomedical correlation lowers the effective cost of edges belonging to the candidate bridge set, thereby reducing the inter-sector accessibility distance. The resulting distance function becomes

(40) $$d_{AB}\left(i,j\right)=\underset{p:i\to j}{\inf}\sum_{e\in p}C_{AB}(e)$$

with corresponding effective sector distance

(41) $$D_{AB}\left(A,B\right)=\frac{1}{\left|A\right|\left|B\right|}\sum_{a\epsilon A}\sum_{b\epsilon B}d_{AB}(a,b)$$

This formulation makes the coupling especially transparent: biomedical correlation directly modulates accessibility cost. It is therefore well suited to graph-based simulations, where one can vary $T\left(A:B\right)$ parametrically and observe the resulting bridge opening and accessibility compression. Although less fundamental than the action-based formulation, it serves as a practical, effective model for numerical exploration.

In medical terms, this rule can be interpreted as follows: when two biomedical sectors are strongly coupled, the effective relational cost of communication, transition, shared pathological expression, or predictive dependence between them is reduced. This does not imply direct anatomical causality, but it provides a mathematical way to represent hidden disease-state proximity.

### 5.6. Motif-Level Biomedical Coupling

The most intrinsic version of the coupling arises when the sectors (A) and (B) are taken to be neighborhoods supporting stable biomedical motifs *H_α_* and *H_β_*. In that setting, the correlation side is quantified by motif-level biomedical coupling

(42) $$T\left(H_{\alpha }:H_{\beta }\right)$$

while the accessibility side is quantified by a bridge gain

(43) $$\Gamma \left(H_{\alpha },H_{\beta };W_{\alpha \beta }\right)$$

The correspondence may then be written as

(44) $$T\left(H_{\alpha }:H_{\beta }\right)↑\;\;\;\;⟹\;\;\;\Gamma \left(H_{\alpha },H_{\beta };W_{\alpha \beta }\right)↑$$

or equivalently,

(45) $$T\left(H_{\alpha }:H_{\beta }\right)↑\;\;\;\;⟹\;\;\;D{(H}_{\alpha },H_{\beta })↓$$

This motif-level formulation is conceptually powerful because it connects the model directly to stable biomedical patterns. Instead of speaking abstractly about two graph regions, one speaks of a correlated pair of disease-relevant motifs whose joint organization is encoded by an accessibility bridge.

For example, a tumor-core radiomic motif and an edema-related motif may become effectively closer as their correlation increases. A respiratory-instability motif and a neurological-outcome motif may become linked through a low-cost disease-state route in an ICU graph. A primary tumor motif and a metastatic niche motif may show bridge-like accessibility compression if their molecular, immune, vascular, or imaging signatures become strongly coupled.

In this sense, the correlation-induced accessibility bridge becomes a bridge between two stable biomedical patterns, not merely between arbitrary graph partitions.

### 5.7. Statistical Existence of Bridge-Supporting Biomedical Configurations

The action-based model also allows one to define the probability that the ensemble occupies a bridge-supporting sector for a given correlated pair. Let

(46) $$\Omega_{B}\left(A,B\right)=\left\{G\in \Omega :\Gamma (A,B;W_{AB})\geq \Gamma_{c}\right\}$$

denote the subset of configurations admitting a biomedical accessibility bridge between A and B. Then the corresponding bridge-supporting probability is

(47) $$P_{B}\left(A,B\right)=\sum_{G\in \Omega_{B}(A,B)}P\left(G|A,B\right)$$

A correlation-induced accessibility-bridge relation is then realized statistically if $P_{B}\left(A,B\right)$ increases monotonically with $T\left(A:B\right)$, or at least exhibits a clear transition once $T\left(A:B\right)$ exceeds the threshold *I*_c_. This formulation is especially useful for numerical and clinical work, because it translates the correspondence into a measurable ensemble-level effect rather than a purely formal relation.

In practical terms, one would test whether increasing biomedical correlation causes the probability of high-gain bridge configurations to increase. If such an effect is robust across graph ensembles, patient subgroups, imaging protocols, and coarse-graining prescriptions, it would support the claim that the model captures a structural correlation–connectivity principle relevant to disease-state organization.

### 5.8. Interpretation of the Biomedical Coupling

The coupling introduced above should be interpreted carefully. It does not claim that correlation alone creates a biological pathway nor that statistical dependence automatically proves causality. Rather, it states that in a biomedical relational substrate where effective disease geometry is inferred from constrained accessibility, strong coarse-grained correlation and bridge-like accessibility compression can be viewed as complementary encodings of the same underlying organization.

The correlation side is a statement about non-factorizable biomedical dependence in the ensemble. The accessibility side is a statement about emergent compression in the induced disease-state metric. The coupling rule identifies when and how these two descriptions coincide.

This interpretation is consistent with the broader relational–informational philosophy. Biological organization, disease states, clinical deterioration, treatment response, and imaging phenotypes may be understood as emergent representational layers of constrained relational structure. The present section extends that logic by treating biomedical correlation and accessibility bridges as two coupled aspects of the same relational substrate. The proposed model is therefore not an external analogy but an internal extension of the principle that informational organization underlies effective biomedical connectivity.

### 5.9. Scope and Limitations of the Present Coupling

It is important to emphasize the scope of the coupling proposed here. The present section does not provide a validated mechanistic model of tumor invasion, metastasis, recurrence, organ failure, treatment resistance, or physiological deterioration. The coupling laws introduced above are structural and effective. Their role is to formalize, within the current biomedical relational framework, the minimal conditions under which strong correlation may be represented as an emergent accessibility bridge.

This limitation is not a weakness but a statement of a theoretical stage. The proposed model is designed to generate mathematically explicit and numerically testable hypotheses about hidden disease connectivity. Any specific biological interpretation of a bridge must be validated using independent clinical, imaging, molecular, longitudinal, interventional, or experimental evidence. Accordingly, the present work should be understood as defining and testing a correlation-induced accessibility-bridge framework at the level presently available to the theory: coarse-grained, graph-based, statistical, and hypothesis-generating.

### 5.10. Summary of Correlation–Accessibility Coupling

This section has introduced the central coupling rules of the paper. The Biomedical Correlation–Compression Principle states the general structural postulate that a stronger non-factorizable biomedical correlation may be represented as reduced effective accessibility distance. This principle was made explicit through the exponential compression ansatz

$$D_{eff}\left(A,B\right)=D_{0}\left(A,B\right)e^{-\alpha T\left(A:B\right)},$$

the threshold correspondence,

$$T\left(A:B\right)\geq I_{c}⟹\Gamma (A,B;W_{AB})\geq \Gamma_{c}$$

and the action-based ensemble formulation

$$S_{B}\left(G;A,B\right)=S_{0}\left(G\right)-\eta T\left(A:B\right)+\mu \sum_{e\in W_{AB}}\Phi (C\left(e\right)).$$

We also introduced the correlation-dependent bridge-cost model, the motif-level realization of the coupling, and the ensemble-level probability $P_{B}\left(A,B\right)$ for bridge-supporting configurations. Together, these definitions provide an explicit mathematical formulation of the correlation-induced accessibility-bridge model within the biomedical relational–informational framework. The next section implements this coupling in numerical proof-of-concept simulations designed to test whether increasing biomedical correlation produces measurable accessibility compression and bridge formation in graph ensembles.

## 6. Numerical Proof-of-Concept Simulation Protocol

The formal developments of the previous sections define a correlation-induced accessibility-bridge model within the biomedical relational–informational framework by coupling coarse-grained biomedical correlation to emergent accessibility compression. The purpose of the present section is to show that this coupling can be implemented explicitly in graph-based models and tested numerically. Our aim is not to reproduce a specific biological mechanism nor to simulate a complete clinical disease process, but to provide a proof of concept: we seek to determine whether increasing non-factorizable biomedical correlation between two sectors can produce a measurable reduction in effective accessibility distance and enhance the probability of high-gain biomedical bridge formation.

The simulations implemented here are deliberately minimal. They are designed to remain faithful to the primitive objects of the framework—weighted biomedical relational graphs, emergent accessibility distance, coarse-grained sectors, and statistical weighting by informational action—while adding the new ingredients required for the present medical construction: sector-level mutual information, candidate biomedical bridge sets, and correlation-dependent accessibility compression. In this sense, the numerical study functions as a consistency check and proof-of-concept implementation of the theory rather than as a detailed mechanistic disease model.

### 6.1. Simulation Objectives

The numerical study is organized around four concrete questions:

1. Correlation–distance relation:

Does increasing biomedical correlation $T\left(A:B\right)$ reduce the effective accessibility distance *D*(*A*,*B*) between sectors *A* and *B*?

2. Bridge emergence:

Does increasing $T\left(A:B\right)$ enhance the formation of high-gain bridge sets *W_AB_* satisfying the biomedical accessibility-bridge criterion?

3. Threshold behavior:

Is there a correlation scale *I_c_* above which bridge-like accessibility compression becomes statistically favored?

4. Robustness:

Do the observed effects persist across graph size, topology, bridge architecture, and coarse-graining choices?

These questions correspond directly to the formal structure of Section 3, Section 4 and Section 5. A positive answer to them would support the claim that the framework realizes a correlation-induced accessibility-bridge principle at the level of explicit biomedical graph ensembles.

### 6.2. Graph Ensembles and Baseline Biomedical Relational Structure

We consider ensembles of weighted undirected graphs $G=\left(V,E,C\right)$ with |V| = N ranging from modest toy values to larger finite systems. The use of undirected graphs in the present section is a simplifying choice made for numerical transparency. The essential bridge mechanism does not depend on undirected symmetry, although directed extensions will be important in future work concerned with asymmetric disease influence, temporal progression, causal inference, or treatment response.

Each graph is generated from a connected random or modular ensemble, depending on the test case, following standard graph-theoretical modeling practice. Examples include the following:

- Erdős–Rényi graphs with enforced connectivity;
- two-community graphs with sparse inter-community background links;
- modular graphs containing planted biomedical motifs or planted bridge candidates.

Each edge *e* ∈ *E* is assigned a strictly positive baseline constraint cost

$$C_{0}\left(e\right)>0$$

drawn from a prescribed distribution such as uniform, exponential, or log-normal. Strict positivity guarantees well-defined shortest-path accessibility distances and avoids trivial zero-cost degeneracies. The emergent node-level accessibility distance is then computed from

$$d\left(i,j\right)=\underset{p:i\to j}{\inf}\sum_{e\epsilon p}C_{0}(e)$$

and the induced coarse-grained sector distance is

$$D\left(A,B\right)=\frac{1}{\left|A\right|\left|B\right|}\sum_{a\epsilon A}\sum_{b\epsilon B}d(a,b)$$

To create a setting in which bridge emergence can be meaningfully studied, the baseline ensemble is chosen so that sectors A and B are initially separated by an appreciable accessibility cost. In modular graph realizations, this is achieved by assigning dense, low-cost internal connectivity within each sector and relatively sparse, higher-cost background connections between them. This produces a well-defined non-bridge baseline against which correlation-induced compression can be measured.

In biomedical terms, the two sectors may be interpreted as two disease-relevant domains that are internally coherent but initially only weakly connected. Examples include tumor core and peritumoral edema, respiratory and neurological domains in critical care, imaging and biomarker modules, primary tumor and metastatic niche, or two organ-system compartments.

**Baseline Parameters Used for the Representative Simulations**

For the representative numerical results reported in Section 7, we used weighted undirected two-sector modular graph ensembles with identical generating parameters across the four modulation regimes. Each graph realization consisted of two internally connected sectors A and B, sparse background inter-sector connectivity, and a planted candidate bridge set *W_AB_*. The principal simulation parameters are summarized in Table 4.

The purpose of this ensemble is not to represent a unique biological system or a complete clinical dataset, but to test whether the proposed coupling produces a bridge-specific effect distinguishable from natural null alternatives. The exact numerical values are therefore less important than the ordering and robustness of the observables across the four modulation regimes.

Because the representative simulations use a planted bridge set, the results should be interpreted as testing the behavior of a specified correlation–compression mechanism rather than as demonstrating fully automatic bridge discovery.

### 6.3. Definition of Coarse-Grained Biomedical Sectors

Two sector types are considered in the simulations.

#### 6.3.1. Region-Based Sectors

In the simplest implementation, A and B are two disjoint node subsets chosen as graph communities or planted modules. These sectors are large enough to admit coarse observables and small enough to remain distinct under averaging. In biomedical terms, such sectors may represent two imaging regions, two organ-system domains, two biomarker modules, or two disease-state compartments.

#### 6.3.2. Motif-Based Sectors

In the more intrinsic implementation, A and B are defined as neighborhoods surrounding planted stable subgraphs *H_α_* and *H_β_*, which play the role of biomedical motifs. This setting is closer to disease-pattern modeling, since the correlation and bridge observables then refer to structured biomedical objects rather than arbitrary graph partitions.

Both sector choices are retained because they test complementary aspects of the theory: region-based sectors show the generic mechanism, while motif-based sectors show that the mechanism survives when correlation is associated with localized, stable biomedical structures.

### 6.4. Coarse-Grained Sector Variables and Biomedical Correlation

To define the correlation-side observable numerically, each sector is assigned a discrete or continuous coarse-grained state variable summarizing its biomedical relational organization. Depending on the simulation mode, examples include the following:

- mean internal accessibility;
- mean bridge occupancy;
- local cost histogram summary;
- motif-presence indicator;
- low-dimensional embedding of sector statistics;
- radiomic feature summary;
- biomarker-module activity;
- physiological instability score.

Given these sector variables *x_A_* and *x_B_*, one estimates the joint distribution *P*(*x_A_*,*x_B_*) empirically over an ensemble of graph realizations or over Monte Carlo samples drawn from a given graph ensemble. The primary biomedical correlation measure is then the mutual information

$$T\left(A:B\right)=\sum_{x_{A},x_{B}}P\left(x_{A},x_{B}\right)\log\frac{P\left(x_{A},x_{B}\right)}{P\left(x_{A}\right)P\left(x_{B}\right)}$$

In simulations where continuous variables are used, the corresponding discretized or kernel-estimated version of mutual information may be employed. In practice, two numerical strategies are especially convenient:

Externally controlled correlation: one imposes a tunable coupling parameter (\rho) that directly correlates sector variables, then measures the resulting $T\left(A:B\right)$;
Action-induced correlation: one samples graphs from the coupled ensemble defined in Section 5 and allows the correlation structure to arise endogenously from the action.

The first strategy is simpler and more transparent for proof-of-concept figures. The second is more intrinsic and closer to the logic of the theory.

In the representative simulations reported below, $T\left(A:B\right)$ is treated as an externally controlled biomedical correlation proxy rather than as an independently estimated clinical correlation from real patient data. This choice isolates the accessibility component of the model: it allows one to test whether increasing non-factorizable correlation strength, once represented at the coarse-grained level, produces bridge-specific accessibility compression. Endogenous estimation of $T\left(A:B\right)$ from sampled biomedical relational states is left as a refinement of the action-based ensemble model and as a future step for patient-derived datasets.

### 6.5. Candidate Biomedical Bridge Architecture

To probe the accessibility side, a candidate bridge set *W_AB_* ⊆ *E* is specified or allowed to emerge between sectors A and B. Two bridge models are considered.

#### 6.5.1. Planted Bridge Model

A small subset of edges or short relay chains connecting A and B is designated in advance as the candidate bridge architecture. Their effective costs are then modulated according to the coupling rule introduced in Section 5. This model is especially useful for visual demonstration, since it provides a clear bridge geometry whose opening and closing can be tracked directly.

In biomedical interpretation, the planted bridge may represent a hypothesized coupling pathway, such as an imaging–biomarker link, a tumor–edema interface, a vascular or immune-mediated route, or a physiological connection between organ systems.

#### 6.5.2. Emergent Bridge Search

Rather than fixing *W_AB_* a priori, one identifies it a posteriori as the subset of edges whose removal produces the largest increase in inter-sector distance. This model is computationally heavier but conceptually stronger, since it allows the bridge structure to be discovered from the accessibility geometry itself rather than assumed in advance.

In both cases, the background distance after bridge removal is computed as

$$D_{bg}\left(A,B;W_{AB}\right)=\frac{1}{\left|A\right|\left|B\right|}\sum_{a\epsilon A}\sum_{b\epsilon B}d_{G\backslash W_{AB}}(a,b)$$

and the bridge gain is

$$\Gamma \left(A,B;W_{AB}\right)=\frac{D_{bg}\left(A,B;W_{AB}\right)}{D\left(A,B\right)}$$

### 6.6. Numerical Implementation of the Coupling Rules

The theoretical coupling rules introduced in Section 5 were implemented numerically through controlled bridge-cost modulation. The purpose of this subsection is to specify how the coupling rules were used in the simulations, how the correlation proxy was swept, how the thresholds may be calibrated, and how null models were constructed. This separation keeps Section 5 focused on the theoretical correspondence and places the implementation details in the numerical protocol.

#### 6.6.1. Effective Compression Model

The representative simulations used the graph-level bridge-cost rule introduced in Section 5.5. For bridge edges belonging to the candidate bridge set $W_{AB}$, the cost was updated according to

$$C_{AB}\left(e\right)=\frac{C_{0}(e)}{1+\alpha T\left(A:B\right)},\;\;\;\;\;\;\;e\in W_{AB}$$

For all non-bridge edges, the baseline cost was preserved:

$$C_{AB}\left(e\right)=C_{0}(e),\;\;\;\;\;\;\;e\notin W_{AB}$$

In this implementation, $T\left(A:B\right)$ was treated as an externally controlled biomedical correlation proxy. It was swept over the grid

$$T\left(A:B\right)=0,0.1,0.2,\ldots ,2.0$$

At each value of $T\left(A:B\right)$, the bridge-edge costs were updated, shortest-path distances were recomputed, and the effective inter-sector distance D(A,B), bridge gain $\Gamma \left(A,B;W_{AB}\right)$, and fractional compression $\Delta_{B}\left(A,B;W_{AB}\right)$ were recorded.

The effective sector distance was computed as

$$D\left(A,B\right)=\frac{1}{\left|A\right|\left|B\right|}\sum_{a\in A}\sum_{b\in B}d(a,b)$$

The bridge gain was computed as

$$\Gamma \left(A,B;W_{AB}\right)=\frac{D_{bg}\left(A,B;W_{AB}\right)}{D(A,B)}$$

where $D_{bg}\left(A,B;W_{AB}\right)$ denotes the background inter-sector distance after removal of the candidate bridge set $W_{AB}$.

The fractional accessibility compression was computed as

$${∆}_{B}\left(A,B;W_{AB}\right)=1-\frac{D(A,B)}{D_{bg}\left(A,B;W_{AB}\right)}$$

This implementation isolates the accessibility effect of the proposed coupling rule: increasing $T\left(A:B\right)$ lowers the cost of the candidate bridge edges and tests whether this produces a measurable reduction in inter-sector accessibility distance.

#### 6.6.2. Action-Based Ensemble Extension

The action-based model introduced in Section 5.4 was retained as the natural ensemble-level extension of the same mechanism. In that formulation, the bridge-coupled action is

$$S_{B}\left(G;A,B\right)=S_{0}\left(G\right)-\eta T\left(A:B\right)+\mu \sum_{e\in W_{AB}}\Phi (C\left(e\right))$$

with corresponding Gibbs-type distribution

$$P\left(G|A,B\right)=\frac{1}{Z_{AB}}e^{-\beta S_{B}(G;A,B)}$$

Strictly speaking, $T\left(A:B\right)$ is an ensemble-level correlation measure induced by the probability distribution over the coarse-grained sector variables. Therefore, the action-based formulation should be read as an effective or mean-field ensemble model unless a graph-level correlation score is specified explicitly.

Sampling from this ensemble may be implemented through Markov-chain updates of edge costs, bridge occupancy, or local rewiring. In the present proof-of-concept study, however, the deterministic bridge-cost modulation was used as the primary numerical engine because it makes the correlation–compression effect and the null-model comparison transparent.

#### 6.6.3. Threshold Calibration and Sensitivity Analysis

The thresholds $I_{c}$ and $\Gamma_{c}$ were treated as operational, ensemble-dependent quantities rather than universal constants. The correlation threshold $I_{c}$ can be calibrated from a permutation-based null distribution of $T\left(A:B\right)$, for example

$$I_{c}\left(A,B\right)=Q_{0.95}\left[T_{null}(A:B)\right]$$

Similarly, the bridge gain threshold $\Gamma_{c}$ can be calibrated from a matched graph-null distribution of bridge gains, for example

$$\Gamma_{c}\left(A,B;W_{AB}\right)=Q_{0.95}\left[\Gamma_{null}\left(A,B;W_{AB}\right)\right]$$

In the representative simulations, the aim was not to select a single clinically optimized cutoff. Instead, D(A,B), $\Gamma \left(A,B;W_{AB}\right)$, and $\Delta_{B}\left(A,B;W_{AB}\right)$ were evaluated across the full correlation-proxy grid. Sensitivity was assessed by repeating the simulations across 80 graph realizations and by comparing the true bridge-coupling regime with matched null regimes generated under the same baseline graph parameters.

#### 6.6.4. Null Models and Edge-Modulation Controls

Four modulation regimes were compared.

First, in the true bridge-coupling regime, only the planted bridge edges in $W_{AB}$ were modulated according to

$$C_{AB}\left(e\right)=\frac{C_{0}(e)}{1+\alpha T\left(A:B\right)},$$

Second, in the no-coupling regime, all edge costs remained equal to their baseline values $C_{0}(e)$, independently of $T\left(A:B\right)$.

Third, in the random non-bridge modulation regime, the same number of non-bridge edges as in $W_{AB}$ was randomly selected and modulated using the same cost rule.

Fourth, in the random inter-sector modulation regime, the same number of inter-sector edges was randomly selected and modulated, without requiring that they belong to the planted bridge set.

For each regime, the same output metrics were computed: effective inter-sector distance D(A,B), bridge gain $\Gamma \left(A,B;W_{AB}\right)$, fractional compression $\Delta_{B}\left(A,B;W_{AB}\right)$, and summary statistics across graph realizations. A bridge-specific effect was considered present when the true bridge-coupling regime produced stronger distance reduction and higher bridge gain than the no-coupling, random non-bridge, and random inter-sector controls.

#### 6.6.5. Effect Estimator and Statistical Identifiability

To make the bridge-specific effect statistically explicit, we define the null-relative compression estimator as

$${\hat{∆}}_{D}^{\left(m\right)}\left(T\right)=\frac{1}{R}\sum_{r=1}^{R}\left[D_{null,m}^{\left(r\right)}\left(A,B;T\right)-D_{bridge}^{\left(r\right)}\left(A,B;T\right)\right]$$

where *R* is the number of graph realizations, *m* denotes the null regime, $D_{bridge}^{\left(r\right)}\left(A,B;T\right)$ is the effective inter-sector distance under true bridge coupling in realization *r*, and $D_{null,m}^{\left(r\right)}\left(A,B;T\right)$ is the corresponding distance under null model *m*. With this sign convention, positive values indicate stronger compression under true bridge coupling than under the null model.

Analogous estimators can be defined for bridge gain and fractional compression:

$${\hat{∆}}_{\Gamma }^{\left(m\right)}\left(T\right)=\frac{1}{R}\sum_{r=1}^{R}\left[\Gamma_{bridge}^{\left(r\right)}\left(A,B;W_{AB};T\right)-\Gamma_{null,m}^{\left(r\right)}\left(A,B;W_{AB};T\right)\right]$$

$${\hat{∆}}_{\Delta_{B}}^{\left(m\right)}\left(T\right)=\frac{1}{R}\sum_{r=1}^{R}\left[\Delta_{B,bridge}^{\left(r\right)}\left(A,B;W_{AB};T\right)-\Delta_{B,null,m}^{\left(r\right)}\left(A,B;W_{AB};T\right)\right]$$

In the present finite proof-of-concept study, identifiability is assessed empirically by comparing these estimators across repeated graph realizations and null regimes. Bootstrap confidence intervals or permutation tests across graph realizations may be used to quantify uncertainty. We do not claim a large-deviation or large-system concentration theorem in the present manuscript. Establishing conditions under which $P\left(\Delta_{D}>0\right)\to 1$ would require additional assumptions on graph generation, edge-cost distributions, dependence structure, noise, and heterogeneity and is left for future theoretical work.

### 6.7. Observables

The simulations track the following observables:

1. Biomedical correlation
  $$T\left(A:B\right)$$
2. Effective inter-sector distance
  $$D\left(A,B\right)$$
3. Bridge gain
  $$\Gamma \left(A,B;W_{AB}\right)$$
4. Fractional compression
  $${∆}_{B}\left(A,B;W_{AB}\right)=1-\frac{D(A,B)}{D_{bg}\left(A,B;W_{AB}\right)}$$
5. Bridge-supporting probability
  $$P_{B}\left(A,B\right)=\sum_{G\in \Omega_{B}(A,B)}P\left(G|A,B\right)$$
6. Bridge response under cost modulation
  $$R_{\lambda }=-\frac{\partial F}{\partial \lambda }$$
7. Optional curvature signature
  $${∆\kappa }_{AB}=\kappa_{W_{AB}}-\kappa_{ext}$$

These observables allow one to test both the direct accessibility effect of correlation and the ensemble-level preference for bridge-supporting configurations.

The representative results reported in Section 7 focus on the first three accessibility-side observables, *D*(*A*,*B*), Γ(*A*,*B*;*W_AB_*), and Δ_B_(*A*,*B*;*W_AB_*). The remaining quantities define natural extensions for action-based, longitudinal, or curvature-sensitive variants of the model.

### 6.8. Simulation Protocol

**Figure 1:**
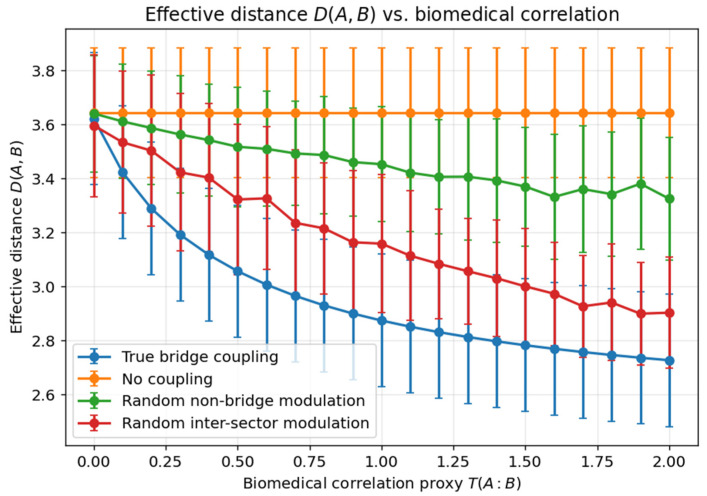
Effective inter-sector distance versus biomedical correlation. Mean effective inter-sector accessibility distance *D*(*A*,*B*) as a function of the biomedical correlation proxy $T\left(A:B\right)$, averaged over 80 random graph realizations. Error bars indicate one standard deviation across realizations. The true bridge-coupling model exhibits the strongest monotonic decrease in *D*(*A*,*B*), while the no-coupling control remains flat. Random non-bridge modulation produces a weaker reduction, and random inter-sector modulation yields an intermediate effect. This shows that accessibility compression is strongest when correlation is coupled specifically to the designated bridge architecture.

**Figure 2:**
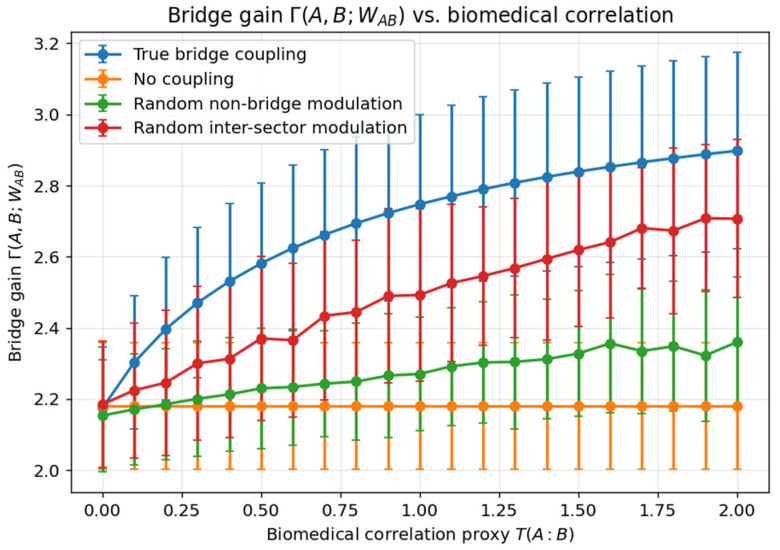
Bridge gain versus biomedical correlation. Mean bridge gain Γ(*A*,*B*;*W_AB_*) as a function of the biomedical correlation proxy $T\left(A:B\right)$, averaged over 80 random graph realizations. Error bars indicate one standard deviation. The true bridge-coupling model produces the largest increase in gain, indicating that the designated bridge set becomes progressively more effective as an accessibility shortcut relative to the background graph with the bridge removed. The no-coupling control remains flat, while the two null models produce weaker responses.

**Figure 3:**
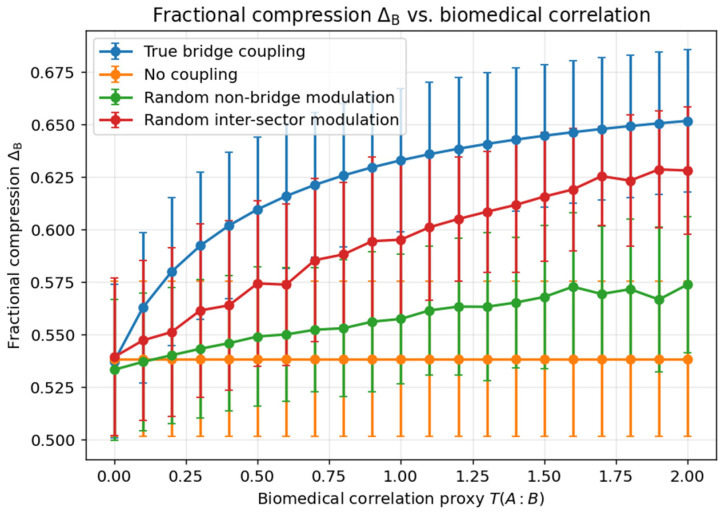
Fractional accessibility compression versus biomedical correlation. Mean fractional compression Δ_B_ as a function of the biomedical correlation proxy $T\left(A:B\right)$, averaged over 80 random graph realizations. Error bars indicate one standard deviation. The true bridge-coupling model shows the largest increase in normalized compression, followed by the random inter-sector control and then the random non-bridge control, while the no-coupling case remains constant. The consistent ordering across models reinforces the interpretation that the accessibility-side response is strongest when correlation acts directly on the designated bridge set.

**Figure 4:**
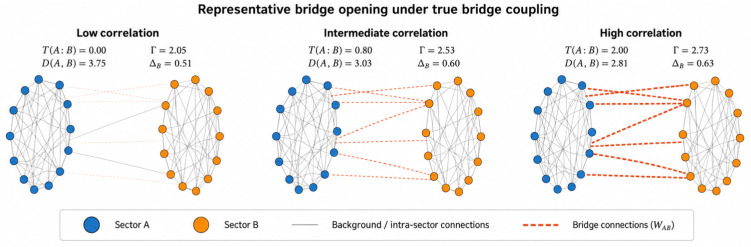
Visual illustration of correlation-induced accessibility-bridge opening in a representative two-sector biomedical graph. The three panels use the same node layout and the same baseline graph realization, while the inter-sector correlation proxy $T\left(A:B\right)$ increases from left to right. Sector A and sector B are shown with distinct colors, background edges are shown in gray, and the candidate bridge edges *W_AB_* are highlighted in red/orange. Bridge-edge width increases as effective accessibility improves under stronger correlation-dependent cost modulation. The (left) panel shows the low-correlation regime, in which the bridge is effectively closed or weak. The (middle) panel shows an intermediate-correlation regime, in which a partial accessibility bridge is visible. The (right) panel shows the high-correlation regime, in which the bridge is maximally open and the effective inter-sector accessibility distance is strongly reduced. Each panel also reports the corresponding effective inter-sector distance D(A,B), bridge gain $\Gamma \left(A,B;W_{AB}\right)$, and fractional compression ${∆}_{B}\left(A,B;W_{AB}\right)$.

The representative simulation sweep used for Figure 1, Figure 2, Figure 3 and Figure 4 proceeded as follows:

1. Generate a baseline connected weighted graph *G*_0_ with sectors *A* and *B*.
2. Compute the background accessibility observables *D*_0_(*A*,*B*) and, when applicable, identify a candidate bridge set *W_AB_*.
3. Assign an externally controlled biomedical correlation proxy $T\left(A:B\right)$ over the grid 0, 0.1, …, 2.0.
4. Update bridge costs or sample the coupled action accordingly.
5. Recompute the accessibility distance *D*(*A*,*B*), bridge gain Γ(*A*,*B*;*W_AB_*), and any auxiliary observables.
6. Repeat the sweep over the selected parameter grid in $T\left(A:B\right)$ and across 80 graph realizations.
7. Average results over multiple graph realizations and random seeds.

Four modulation regimes were evaluated using independent randomized graph ensembles generated from the same parameter specification: true bridge coupling, no coupling, random non-bridge modulation, and random inter-sector modulation. This design tests whether the bridge-specific effect persists at the ensemble level while separating it from both the trivial absence of modulation and the generic cost reduction elsewhere in the graph.

The main plots of interest are:

- *D*(*A*,*B*) versus $T\left(A:B\right)$,
- Γ(*A*,*B*;*W_AB_*) versus $T\left(A:B\right)$,
- *P*_B_(*A*,*B*) versus $T\left(A:B\right)$,
- accessibility heat maps before and after bridge opening,
- graph visualizations showing the opening of low-cost bridge sets.

These provide both quantitative and intuitive support for the correlation-induced accessibility-bridge mechanism.

### 6.9. Expected Numerical Signatures

If the framework implements the intended biomedical correspondence, one expects the following numerical signatures:

1. Monotonic compression: the effective inter-sector distance *D*(*A*,*B*) decreases as $T\left(A:B\right)$ increases;
2. Bridge amplification: the gain factor Γ(*A*,*B*;*W_AB_*) increases with correlation strength;
3. Threshold-compatible behavior: once $T\left(A:B\right)$ exceeds a regime *I_c_*, the probability of bridge-supporting configurations may increase, providing a natural extension of the monotonic compression analysis;
4. Bridge localization: accessibility compression is mediated by a bounded candidate bridge set rather than by diffuse graph-wide change;
5. Robustness across ensembles: the qualitative effect persists under variation in graph topology, cost distribution, bridge architecture, and coarse-graining.

The observation of these signatures would not prove a specific biological mechanism or clinical causal pathway. It would, however, support the claim that the present biomedical relational framework realizes a structural correlation–connectivity principle in explicit numerical models.

### 6.10. Minimal Toy Example

To illustrate the mechanism transparently, one may consider a minimal two-cluster biomedical toy model. Let A and B be two internally well-connected sectors linked only by sparse, high-cost background edges. Introduce a small planted bridge set *W_AB_* consisting of a few designated low-cost cross-links. Then define a correlation variable between the sectors and let the bridge-edge cost obey

$$C_{AB}\left(e\right)=\frac{C_{0}(e)}{1+\alpha T\left(A:B\right)},\;\;\;\;\;\;e\;\epsilon \;W_{AB}$$

As $T\left(A:B\right)$ increases, the bridge edges become more accessible, and the mean inter-sector distance

$$D\left(A,B\right)=\frac{1}{\left|A\right|\left|B\right|}\sum_{a\epsilon A}\sum_{b\epsilon B}d(a,b)$$

decreases accordingly.

The graph may then be visualized as two biomedical sectors becoming progressively closer in emergent accessibility geometry despite no necessary change in anatomical distance. This toy model is especially useful for figures and animations illustrating the basic idea. For example, it may represent two imaging-defined regions, two biomarker modules, or two physiological domains whose effective disease-state separation decreases as their coupling increases.

### 6.11. Reproducibility and Computational Scope

The numerical procedures required for the present section are algorithmically straightforward and do not rely on specialized physical or biological solvers. Emergent accessibility distances are computed using standard shortest-path methods. Mutual information may be estimated by standard discrete or continuous estimators. Gibbs-type ensembles can be sampled with conventional Markov-chain Monte Carlo updates on edge costs and local graph structure. Bridge identification may be performed through direct deletion tests, path-centrality statistics, or edge-removal sensitivity scans. In this sense, the simulation program is fully reproducible using standard graph-analysis and statistical-inference libraries.

At the same time, the present simulations are not intended to establish biological universality, clinical validity, or patient-level predictive performance. Their role is proof-of-concept: to demonstrate that the formal coupling rules introduced in the previous sections generate the expected bridge-like accessibility compression in explicit finite ensembles. Application to real medical datasets will require additional steps, including cohort definition, feature harmonization, validation, biological interpretation, and external testing.

### 6.12. Interpretation of the Numerical Program

The significance of the simulation program is conceptual as well as technical. In many biomedical studies, correlations are identified and used predictively without reconstructing the effective accessibility geometry through which disease sectors become connected. Conversely, network models may define connectivity without explicitly linking it to non-factorizable inter-sector dependence. The present numerical study addresses this gap in the simplest possible way.

If a strong biomedical correlation in a graph-based relational substrate is sufficient to generate emergent accessibility bridges with high-gain properties, then the structural relation between correlation and hidden disease connectivity may be more general than any single disease mechanism or modality-specific model. This does not mean that the graph models reproduce tumor invasion, metastasis, organ failure, recurrence, or treatment response in full biological detail. They do not. What they show, if successful, is that a correlation–accessibility mechanism can arise already at the level of abstract biomedical relational organization.

That is the specific claim the present paper is designed to test.

### 6.13. Summary of the Numerical Strategy

This section has specified the numerical strategy used to test the proposed framework. Weighted graph ensembles are used to represent biomedical relational configurations, coarse-grained sectors are defined as graph regions or motif neighborhoods, and biomedical correlation is quantified through mutual-information-type coupling. In the representative simulations, candidate bridge sets are planted to test the correlation–compression mechanism directly, while operational bridge discovery is left as a natural extension. Bridge gain, fractional accessibility compression, and related ensemble-level quantities provide the main numerical signatures of correlation-induced accessibility-bridge behavior. With this simulation architecture in place, the next section presents the numerical results and their interpretation.

## 7. Representative Numerical Results and Interpretation

The simulations described in Section 6 were designed to test whether the proposed biomedical correlation–accessibility coupling produces a bridge-specific reduction in effective inter-sector distance, and whether this effect remains distinguishable from suitable null models. The numerical results support this claim at the level of a proof-of-concept ensemble study. Across 80 randomized graph realizations, the model in which biomedical correlation modulates the designated bridge set *W_AB_* exhibited the strongest monotonic decrease in effective inter-sector distance *D*(*A*,*B*), the strongest increase in bridge gain Γ(*A*,*B*;*W_AB_*), and the largest increase in fractional compression Δ_B_.

By contrast, the no-coupling control remained flat, while random non-bridge modulation produced only modest changes. Random inter-sector modulation generated an intermediate effect, as expected from the generic shortening of cross-sector accessibility under reduced inter-sector costs, but remained consistently weaker than the designated bridge-coupling model. Taken together, these results indicate that the observed accessibility compression is not merely a trivial consequence of arbitrary edge-cost reduction but is enhanced when correlation is coupled specifically to a bridge architecture linking the two biomedical sectors.

Figure 1, Figure 2 and Figure 3 show that the true bridge-coupling model exhibits the strongest monotonic accessibility-side response across all principal observables. Effective inter-sector distance decreases most strongly, while bridge gain and fractional compression increase most strongly, under correlation-dependent modulation of the designated bridge set. The no-coupling control remains flat, confirming that the effect is not an artifact of averaging or visualization. Random non-bridge modulation produces weaker changes, whereas random inter-sector modulation generates an intermediate response, indicating that generic cross-sector cost reduction can shorten accessibility but does so less efficiently than the designated bridge architecture. Figure 4 provides representative graph realizations illustrating the progressive opening of the bridge under increasing biomedical correlation proxy values.

To complement the graphical trends shown in Figure 1, Figure 2, Figure 3 and Figure 4, Table 5 summarizes the principal effect sizes obtained across the four modulation regimes. The reported values quantify the net change in effective inter-sector distance, bridge gain, and fractional compression between the lowest and highest correlation-proxy conditions. This tabular summary provides a compact comparison of the accessibility response induced by true bridge coupling, no coupling, random non-bridge modulation, and random inter-sector modulation. The strongest reduction in effective distance and the largest increases in bridge gain and fractional compression are observed under true bridge coupling, supporting the interpretation that correlation-dependent modulation is most effective when it acts on the designated biomedical bridge architecture.

The consistent ordering of all three observables supports the interpretation that accessibility compression is strongest when correlation acts on a structurally privileged biomedical bridge set rather than on arbitrary non-bridge or generic inter-sector edges.

Because the simulations were repeated across 80 randomized graph realizations, the qualitative ordering of modulation regimes should be interpreted together with the corresponding mean trajectories and variability bands shown in Figure 1, Figure 2 and Figure 3. The effect-size table summarizes the endpoint differences, whereas the figures provide the ensemble-level monotonic trends.

Formal inferential comparisons are not treated as clinical hypothesis tests here, because the simulations are synthetic proof-of-concept ensembles rather than patient-derived samples.

### 7.1. Effective Distance Decreases Most Strongly Under True Bridge Coupling

The most direct accessibility-side observable is the effective inter-sector accessibility distance *D*(*A*,*B*). In the true bridge-coupling model, *D*(*A*,*B*) decreased from approximately 3.62 at $T\left(A:B\right)=0$ to approximately 2.73 at $T\left(A:B\right)=2.0$, corresponding to a net change of −0.896. This was the largest distance reduction among all tested models. The no-coupling control showed no change, as expected. Random non-bridge modulation produced only a modest reduction, −0.316, while random inter-sector modulation yielded an intermediate reduction, −0.694.

These comparisons demonstrate that the main effect is not an artifact of the plotting pipeline or of ensemble averaging, since the control without coupling remains exactly flat. More importantly, they show that the designated bridge set is the most efficient target for accessibility compression within the tested graph architecture.

The difference between the true bridge model and the random inter-sector null is especially informative. Since random inter-sector modulation also lowers selected cross-sector costs, it is expected to shorten the effective distance to some extent. The fact that it does so, yet still remains systematically weaker than the planted bridge-coupling model, indicates that the effect depends not only on lowering inter-sector constraints in general but also on lowering them in a structurally privileged subset that functions as a high-impact bridge. This is precisely the kind of bridge-specificity required for a biomedical accessibility-bridge interpretation.

### 7.2. Bridge Gain Confirms the Structural Specificity of the Effect

The bridge gain

$$\Gamma \left(A,B;W_{AB}\right)=\frac{D_{bg}\left(A,B;W_{AB}\right)}{D\left(A,B\right)}$$

provides the clearest measure of accessibility compression relative to the background graph with the candidate bridge removed. In the true bridge-coupling model, the gain increased from approximately 2.17 to 2.90, a net increase of +0.723. This exceeded the gain increase observed in the random inter-sector null, +0.522, and was substantially larger than the increase produced by random non-bridge modulation, +0.207. The no-coupling control again remained flat.

This result is arguably the strongest numerical support for the paper’s central claim. While reduced effective distance alone could in principle arise from many forms of cost redistribution, bridge gain measures the accessibility advantage conferred by the candidate bridge architecture relative to the background. The fact that gain increases most strongly when correlation is coupled directly to the designated bridge set supports the interpretation that the model is realizing a bridge-specific biomedical accessibility structure rather than merely redistributing cost in a diffuse or nonspecific way.

### 7.3. Fractional Compression Shows Consistent Accessibility-Side Behavior

The fractional compression observable

$${∆}_{B}\left(A,B;W_{AB}\right)=1-\frac{D(A,B)}{D_{bg}\left(A,B;W_{AB}\right)}$$

provides a normalized measure of the same accessibility effect. In the true bridge-coupling model, Δ**_B_** increased from approximately 0.537 to 0.652, corresponding to a net change of +0.114. This again exceeded the increase produced by the random inter-sector null, +0.089, and was much larger than that produced by random non-bridge modulation, +0.041. The no-coupling control remained unchanged.

The consistency of this ordering across all three main observables—effective distance, bridge gain, and fractional compression—is important. If only one observable favored the true bridge model while others did not, the interpretation would be fragile. Instead, all principal accessibility-side metrics rank the models in the same order:

**true bridge coupling > random inter-sector modulation > random non-bridge modulation > no coupling**

This repeated ordering substantially strengthens the case that the correlation–accessibility relation is structurally meaningful within the present ensemble.

### 7.4. Visual Graph Realizations Match the Ensemble Trends

Representative graph visualizations are consistent with the quantitative results and provide an intuitive illustration of the bridge-opening mechanism, as shown in Figure 4. As the biomedical correlation proxy $T\left(A:B\right)$ increases, the designated bridge edges thicken in the weighted drawing, reflecting their reduced effective cost, while the measured effective distance between sectors decreases and bridge gain increases. These visualizations are not, by themselves, evidence of the mechanism, but they serve an important interpretive role: they make the accessibility compression process directly visible in a graph setting where no anatomical continuity or predefined causal pathway has been assumed.

This point matters conceptually. The present model does not depict a biological tunnel or a direct mechanistic route. Instead, it shows how a low-cost bridge in a biomedical relational network becomes a dominant accessibility shortcut between coarse-grained sectors. Figure 4 therefore plays the role of an accessibility-side illustration: not a literal anatomical bridge, but a structural bridge in emergent disease-state geometry.

### 7.5. What the Null Models Show

The null models clarify the status of the result. The no-coupling control confirms that the observed effect does not arise in the absence of a correlation-dependent cost rule. The random non-bridge control shows that arbitrary cost reduction elsewhere in the graph has only a weak influence on the inter-sector geometry. The random inter-sector control shows that generic shortening of cross-sector paths can indeed produce a partial bridge-like effect, but not as strongly as modulation of the designated bridge set.

This hierarchy is scientifically useful. It means the true bridge-coupling effect is neither trivial nor absolute. It is not trivial, because arbitrary modulation does not reproduce it equally well. It is not absolute, because alternative inter-sector modulations can still generate weaker accessibility compression. The correct interpretation is therefore not that only one exact bridge architecture can realize the effect but that the designated bridge architecture does so most efficiently and systematically in the tested ensemble. That is a stronger and more nuanced result than a simple all-or-nothing claim.

### 7.6. Interpretation as a Biomedical Correlation–Accessibility Proof of Concept

Within the limits of the present framework, the ensemble results support the intended biomedical interpretation. Increasing biomedical correlation proxy values produce increasing accessibility compression between coarse-grained sectors, and this compression is strongest when correlation is coupled specifically to a bridge architecture linking those sectors. In that sense, the simulations support the idea that strong inter-sector biomedical correlation can be encoded structurally as accessibility compression in a disease-state graph.

At the same time, the results should be interpreted with appropriate caution. The simulations do not derive the coupling law from a specific biological mechanism. They do not establish a causal pathway, a validated clinical biomarker, a mechanistic model of tumor invasion, a metastatic route, a physiological deterioration cascade, or a treatment-response mechanism. What they do establish is a bridge-specific, statistically robust, graph-based realization of the core structural intuition: correlation and effective connectivity can act as complementary descriptions of the same underlying biomedical relational organization.

This is exactly the level of claim appropriate to a proof-of-concept study built on the biomedical relational–informational framework.

In the representative planted-bridge simulations, the candidate bridge architecture is already present at baseline; the numerical experiment therefore demonstrates correlation-dependent bridge opening and strengthening, while automatic bridge discovery or spontaneous bridge creation is left to the emergent-bridge search extension.

### 7.7. Main Quantitative Summary

The principal effect sizes are summarized in Table 5. These values confirm that the true bridge-coupling model produces the strongest accessibility response in all principal observables. The most important result is the consistent ranking of modulation regimes across *D*(*A*,*B*), $\Gamma \left(A,B;W_{AB}\right)$, and ${∆}_{B}$. This agreement suggests that the observed effect is not dependent on a single metric but reflects a coherent bridge-specific response of the graph ensemble.

From a biomedical perspective, the result can be interpreted as follows: when correlation acts on a structurally privileged route between two disease-relevant sectors, the effective disease-state distance between those sectors decreases more efficiently than when similar cost modulation is applied randomly. This provides a mathematical basis for identifying candidate hidden connectivity pathways in patient-specific or cohort-level biomedical networks.

### 7.8. Summary of Numerical Findings

The numerical results show that the proposed coupling generates systematic correlation-dependent accessibility compression, with the strongest effect occurring when correlation modulates the designated bridge set. This response is not reproduced to the same extent by random non-bridge or generic inter-sector null models, indicating that the observed compression is bridge-specific rather than a diffuse consequence of graph perturbation. The increase in bridge gain and fractional compression therefore supports the proposed biomedical correlation–accessibility principle at the level of a relational proof of concept. The next section turns from numerical evidence to broader interpretation, limitations, and future directions.

## 8. Discussion, Limitations, and Future Directions

The present work set out to examine whether hidden biomedical connectivity can be formulated within a relational framework in which effective disease-state geometry is not assumed in advance but instead emerges from constrained accessibility among biomedical variables, tissue regions, imaging features, physiological domains, or disease-relevant motifs. Building on the relational–informational substrate introduced in the foundational framework, we defined the correlation side in terms of robust, non-factorizable dependence between coarse-grained biomedical sectors and the accessibility side in terms of localized, stable compression associated with bridge sets in the emergent disease-state geometry. We then coupled these two structures analytically and tested the resulting mechanism numerically in graph ensembles. The combined results support a clear proof-of-concept conclusion: within the present biomedical relational framework, increasing sector-level correlation can be represented structurally by bridge-specific accessibility compression.

This conclusion matters for two reasons. First, it shows that hidden disease connectivity can be formulated in a language that does not require anatomical proximity, predefined causal pathways, or fixed feature-space adjacency as primitive assumptions. Second, it suggests that the relationship between biomedical correlation and effective connectivity may be more general than the specific clinical, molecular, radiomic, or physiological settings in which it is usually studied. In the present theory, disease-state geometry is understood as a derived representational layer built from constrained biomedical organization. It is therefore natural that sufficiently strong coarse-grained correlation should admit a geometric encoding in the form of reduced effective accessibility distance and bridge formation.

At the same time, the strength of this conclusion must be stated carefully. The present work does not provide a validated mechanistic model of tumor invasion, metastasis, recurrence, organ failure, treatment resistance, radiotherapy response, or physiological deterioration. It does not establish that a graph-theoretic bridge is necessarily an anatomical route, a causal pathway, or a biological mechanism. Nor does it prove that mutual information alone can distinguish direct disease coupling from confounding, shared background effects, cohort structure, or indirect association. What it does provide is a structural realization of a biomedical correlation–accessibility principle at the level currently available to the framework: a statistically defined, coarse-grained, emergent disease-state geometry in which correlation and effective connectivity are coupled within a single relational substrate. This distinction is essential and should be maintained throughout any discussion of the paper’s significance.

### 8.1. Scope and Main Limitations

The present work has established the following:

1. a rigorous accessibility-side notion of biomedical bridge formation can be defined using accessibility compression in a weighted biomedical relational graph;
2. a correlation-side notion of inter-sector biomedical coupling can be defined using mutual-information-type dependence between coarse-grained sectors;
3. the two can be coupled analytically through explicit compression and action-based models;
4. in randomized graph ensembles, this coupling generates bridge-specific accessibility compression stronger than multiple null models.

These are substantive results. They show that the framework is rich enough to support a nontrivial biomedical correlation–accessibility construction and that the resulting mechanism is not empty or tautological.

However, several equally important things have not been established. The present work has not derived the coupling from a specific molecular, physiological, radiomic, or clinical mechanism. It has not demonstrated that the mutual-information-type quantity used here is equivalent to causal influence. It has not shown that a bridge detected in graph space necessarily corresponds to a vascular, neural, immune, metabolic, metastatic, or treatment-response pathway. It has not yet been validated on real patient datasets. Finally, it has not shown that the observed graph-theoretic compression survives across diseases, institutions, scanners, cohorts, modalities, or longitudinal clinical settings. These remain open questions.

For clarity, the main limitations of the present proof-of-concept study can be summarized as follows. First, the framework is theoretical and has not yet been validated on real patient cohorts. Second, mutual-information-type dependence does not by itself establish causality, mediation, or biological mechanism. Third, the proposed accessibility bridges are graph-theoretical structures, not anatomical channels, metastatic routes, vascular pathways, neural tracts, or proven physiological mechanisms. Fourth, the numerical experiments use controlled graph ensembles and specified bridge architectures; future work must include automatic bridge discovery, sensitivity analysis, and validation across graph sizes, disease types, modalities, institutions, and external cohorts. Fifth, clinical translation will require longitudinal data, uncertainty quantification, confounder adjustment, and independent evaluation against diagnostic, prognostic, or mechanistic endpoints.

### 8.2. Conceptual Significance

One of the most important outcomes of the present study is conceptual clarification. In many biomedical studies, correlation, prediction, and connectivity are treated as related but distinct concepts. Predictive models may detect associations without explaining whether the corresponding variables become effectively close in disease-state space. Network models may define connectivity without explicitly linking it to non-factorizable inter-sector dependence. Mechanistic studies may focus on causal pathways but may miss broader relational structure detectable across multimodal data.

The present framework offers a way to connect these layers. The correlation side becomes a statement about non-factorizable biomedical dependence in an ensemble of coarse-grained sector states, while the accessibility side becomes a statement about anomalous compression in the induced disease-state geometry. Under this reinterpretation, the model does not claim that correlation is a mechanism. Rather, it proposes that strong, persistent correlation may identify where effective biomedical distance has been reduced and where hidden connectivity should be investigated.

This is structurally important. It means that the relationship between correlation and disease connectivity need not be treated as an additional interpretive step imposed after statistical analysis. Instead, both may be understood as complementary descriptions of the same relational organization: one statistical, the other geometric. In this sense, the present work extends the relational–informational program into computational medicine, radiomics, systems biology, and disease-network modeling.

### 8.3. Interpretation of the Numerical Results

The numerical results support the proposed framework in a way that is stronger than a single illustrative toy example but weaker than a full clinical validation. Their most important feature is not merely that increasing the biomedical correlation proxy produces reduced effective distance, but that this effect is strongest when correlation is coupled specifically to the designated bridge set. The no-coupling control remains flat, confirming that the observed behavior is not a plotting artifact or a generic feature of the base graph ensemble. Random non-bridge modulation produces only weak changes, showing that arbitrary cost reduction elsewhere in the graph does not reproduce the effect efficiently. Random inter-sector modulation does produce a substantial intermediate response but remains consistently weaker than the true bridge-coupling model. This indicates that the effect is not unique to one exact edge set yet is still structurally enhanced by a bridge architecture that is especially well positioned to compress accessibility between the chosen sectors.

This nuance is important. A weaker interpretation would claim that only the planted bridge can generate bridge-like behavior. That would not be supported by the present results. The more accurate interpretation is that bridge-specific accessibility structures provide the most efficient geometric encoding of the imposed biomedical correlation within the tested ensembles. That is a more modest claim, but also a more credible and scientifically interesting one.

From a medical perspective, this means that not every correlation in a biomedical graph is equally meaningful. Some correlations may reduce effective disease-state distance only weakly or diffusely, whereas others may act through structurally privileged routes that behave like accessibility bridges. Identifying such routes could be useful for hypothesis generation in radiomics, tumor progression, recurrence modeling, organ-system interaction, multimodal prognosis, and patient-specific disease-network analysis.

### 8.4. Dependence on Coarse-Graining

A central issue for future work is the role of coarse-graining. In the present paper, the sectors A and B are defined either as graph regions or as neighborhoods supporting stable biomedical motifs, and the corresponding sector variables are chosen from a class of coarse observables. This is sufficient for proof-of-concept purposes, but the details of the correlation–accessibility relation may depend quantitatively on how coarse-graining is performed. Different partition schemes, motif definitions, imaging regions, biomarker groupings, physiological domains, and sector-state variables may alter both the measured mutual information and the observed accessibility compression.

This dependence should not be viewed as a flaw unique to the present theory. Any emergent biomedical description depends on what is being coarse-grained and at what scale. A tumor may be described at the level of voxels, radiomic features, subregions, histological compartments, molecular pathways, or patient-level outcomes. An ICU patient may be described through individual laboratory values, physiological systems, temporal trajectories, or outcome states. In each case, the relational geometry may change with the scale of description.

The important question is therefore not whether coarse-graining matters but whether the proposed bridge behavior remains stable across reasonable coarse-graining prescriptions. A convincing biomedical application should show at least partial robustness under changes in sector definition, feature selection, imaging segmentation, patient subgroup, and modality. Testing such stability is a natural next step.

### 8.5. Relation to Network Medicine, Radiomics, and Systems Biology

The present work is closely related to network medicine, radiomics, systems biology, and graph-based machine learning, but it is not identical to any one of these fields. Network medicine often begins from known or inferred biological interaction networks, such as gene, protein, pathway, or disease-module graphs. Radiomics extracts high-dimensional quantitative features from medical images and relates them to clinical endpoints. Systems biology studies multiscale interactions among biological components. Graph-based machine learning models relationships among variables, patients, regions, or modalities.

The value of the present framework lies in its specific emphasis on accessibility compression. It does not merely ask whether two biomedical sectors are connected or correlated. It asks whether their correlation is associated with a measurable reduction in effective disease-state distance and whether that reduction is localized through a bridge-like subset of relations. This gives the model a distinctive operational interpretation: hidden connectivity is identified not only by statistical dependence but also by the ability of a candidate bridge set to shorten accessibility between otherwise separated sectors.

This distinction defines the main potential biomedical value of the framework as a hypothesis-generating tool. In radiomics, accessibility bridges could help prioritize candidate hidden links between enhancing tumor, necrosis, edema, peritumoral tissue, radiomic habitats, and recurrence-prone regions. In oncology, they could provide a formal way to describe effective proximity between primary tumor phenotypes, immune microenvironment states, vascular interfaces, treatment-response patterns, and metastatic niches. In critical care, bridge observables could be used to study coupling between respiratory, metabolic, inflammatory, hemodynamic, and neurological sectors. In multimodal prognosis, the framework may help explain why imaging, laboratory, physiological, and clinical variables become jointly predictive when considered as parts of a relational disease-state geometry. These examples are not claimed here as validated applications; rather, they identify concrete biomedical contexts in which bridge gain, accessibility compression, and correlation-dependent bridge modulation could be tested in real datasets.

### 8.6. Causality, Temporality, and Clinical Interpretation

Perhaps the most important limitation of the present work is the absence of an explicit causal and temporal model. The bridges analyzed here are bridges in an accessibility geometry, not yet validated causal pathways or time-directed disease trajectories. This has several consequences. One cannot yet conclude that information, pathology, or biological influence physically travels through a detected bridge. One cannot yet distinguish sharply between direct causation, indirect mediation, common drivers, confounding, and purely statistical dependence. One also cannot yet determine whether bridge opening precedes disease progression, follows it, or merely reflects a cross-sectional signature of disease state.

These are not small gaps; they define the frontier between the current structural theory and a clinically validated model. A major future direction is therefore to combine the present correlation–compression mechanism with directed graphs, longitudinal data, causal inference, time-series modeling, and intervention-sensitive analysis. Only at that stage could one begin to distinguish predictive bridges from mechanistic bridges.

For clinical interpretation, the safest formulation is therefore the following: a biomedical accessibility bridge is a candidate hidden connectivity structure. It may suggest where mechanistic investigation should be focused, but it does not by itself establish a mechanism. This distinction is particularly important if the model is applied to high-stakes medical contexts such as cancer progression, recurrence prediction, ICU prognosis, or treatment planning.

### 8.7. Toward a More Intrinsic Biomedical Interpretation

A second major limitation is the effective status of the correlation measure. Mutual information is a powerful and appropriate observable for the present statistical framework, but it is not by itself a uniquely biological, causal, or mechanistic quantity. In the current paper, biomedical coupling is therefore used in a structural and operational sense rather than as a direct mechanistic claim. This is acceptable for a proof-of-concept theory, but future work should seek more intrinsic biomedical interpretations.

One possible route would be to define sector variables directly from biologically grounded features, such as hypoxia markers, perfusion parameters, immune signatures, molecular pathways, radiomic habitats, or clinically validated physiological indices. Another would be to estimate correlations from longitudinal trajectories, allowing bridge formations to be linked to disease progression or treatment response. A third route would be to combine mutual information with causal discovery, mediation analysis, survival modeling, explainable machine learning, or mechanistic simulations. These developments would considerably strengthen the biomedical interpretation of the model.

### 8.8. Toward Dynamic Bridge Modeling

The present work treats bridge formation primarily through static or quasi-static observables: effective distance, bridge gain, fractional compression, and ensemble weight. A further step is to study the dynamics of bridge emergence, persistence, and decay. This would require following the evolution of candidate bridge sets over time and measuring bridge lifetimes, opening or closing transitions, hysteresis, and response to perturbations.

Such a dynamic extension would be valuable for several reasons. It would allow one to distinguish transient correlations from stable disease connectivity. It would also connect naturally to longitudinal medical data, such as serial MRI, repeated blood-gas measurements, laboratory trajectories, treatment-response monitoring, or progression-free survival follow-up. Finally, it might reveal threshold phenomena in which disease sectors remain weakly connected until a critical correlation level is reached, after which bridge-like accessibility rapidly increases.

In medical terms, dynamic bridge modeling could be particularly relevant for tumor evolution, metastatic transition, recurrence risk, ICU deterioration, treatment resistance, or radiation-induced tissue injury.

### 8.9. Scaling, Real Datasets, and Generalizability

Another important question concerns scaling. The present simulations are finite, graph-based, and deliberately minimal. They demonstrate the mechanism clearly, but they do not yet answer whether the correlation-induced accessibility effect persists, sharpens, or changes qualitatively as system size grows. A natural next step is therefore to examine larger graph ensembles, higher-dimensional biomedical feature spaces, patient-derived networks, and broader bridge architectures while tracking the scaling behavior of the main observables. The present simulations should not be interpreted as establishing a universal scaling law. The exponential expression introduced in Section 5 is a minimal phenomenological ansatz for monotone correlation–compression coupling, whereas the representative numerical simulations use a rational bridge-edge cost modulation. Therefore, the observed curves demonstrate monotone bridge-specific compression, not a fitted universal exponential or power-law dependence. In the current proof-of-concept study, graph size, modularity, and inverse-temperature effects are not systematically varied. A full scaling analysis would require simulations across multiple values of |V|, modularity Q, bridge density, edge-cost distributions, and, for action-based sampling, inverse temperature β. Such analysis is now identified as a necessary next step before claiming any universal scaling behavior.

A related sensitivity analysis should examine whether the observed bridge-specific accessibility compression depends on the particular functional form chosen for the correlation–compression coupling. In addition to the rational bridge-cost rule used in the representative simulations, future numerical tests should compare exponential, rational, linear-threshold, and sigmoidal coupling laws. Such an analysis would help determine whether the main result reflects a general consequence of monotone correlation-dependent accessibility compression, rather than an artifact of a single phenomenological equation.

In particular, one would like to know whether the bridge-specific advantage of the true coupling persists in larger systems, whether the gap between true bridge coupling and null models grows or shrinks with size, and whether the correspondence exhibits any form of robustness across diseases and modalities. In real datasets, additional challenges will arise: missing data, measurement noise, scanner variability, cohort imbalance, treatment heterogeneity, feature collinearity, segmentation variability, and limited sample size.

For this reason, clinical translation should proceed in stages. First, the method should be tested on controlled synthetic and semi-synthetic biomedical graphs. Second, it should be applied retrospectively to well-curated datasets with known clinical endpoints. Third, bridge-derived observables should be evaluated for prognostic, diagnostic, or explanatory value. Finally, the model should undergo external validation across independent cohorts.

### 8.10. Future Directions

The most immediate future directions suggested by the present work are:

1. More intrinsic biomedical sector definitions.

Replace generic region-based sectors with biologically grounded sectors, such as tumor habitats, organ-system domains, biomarker modules, physiological subsystems, or treatment-response compartments.

2. Endogenous biomedical correlation.

Move from externally imposed or proxy-controlled correlation to correlation estimated directly from patient data, longitudinal trajectories, imaging-derived features, or sampled biomedical relational ensembles.

3. Automatic bridge discovery.

Replace planted bridge sets with bridges identified algorithmically from accessibility sensitivity, edge-removal impact, path importance, explainable graph learning, or perturbation analysis. This is especially important because the representative simulations use a specified bridge architecture.

4. Directed and temporal extensions.

Incorporate directed accessibility, temporal ordering, longitudinal measurements, and causal inference to approach disease progression and treatment-response modeling.

5. Biological refinement.

Develop motif-level interpretations grounded in radiomic habitats, molecular pathways, immune microenvironment states, physiological instability patterns, or validated clinical phenotypes.

6. Dynamic bridge studies.

Track opening, persistence, and decay of bridges under explicit disease evolution, treatment perturbation, or clinical deterioration.

7. Large-scale and real-data studies.

Test robustness and generalizability across graph size, topology, imaging modality, disease type, patient subgroup, and external validation cohort.

Taken together, these directions define a coherent research program rather than a loose set of possible extensions. They all address the same central challenge: moving from a structural proof of concept toward a clinically meaningful theory of hidden biomedical connectivity in relational disease-state spaces.

A direct empirical validation of the proposed framework is beyond the scope of the present proof-of-concept study. Applying the model to real biomedical data would require several additional steps: definition of a clinically meaningful cohort, harmonization of imaging, biomarker, physiological, or electronic-health-record variables, construction of patient-level or cohort-level graphs, estimation of sector-level dependence with uncertainty correction, identification of candidate bridge structures, comparison with appropriate null models, and validation against independent clinical endpoints. For this reason, we do not present the present synthetic simulations as clinical validation. Future work should test the framework on radiomics datasets, ICU physiological monitoring cohorts, cancer biomarker networks, recurrence datasets, or multimodal clinical datasets with independent validation. Until such studies are performed, the proposed bridges should be interpreted as hypothesis-generating structural candidates rather than validated clinical biomarkers or treatment rules.

## 9. Conclusions

The present study formalized correlation-induced accessibility bridges as localized, high-gain reductions in effective inter-sector distance within biomedical relational graphs. The main methodological contribution is the explicit coupling of non-factorizable sector-level dependence, quantified by mutual-information-type observables, to graph-based accessibility compression. In this framework, biomedical correlation and hidden disease connectivity are not treated as separate descriptors but as complementary statistical and geometric manifestations of constrained relational organization.

We introduced operational definitions for bridge-relevant correlation, candidate bridge sets, effective inter-sector accessibility distance, bridge gain, and fractional accessibility compression. We also formulated the correlation–compression relation through a monotone effective coupling law, a threshold correspondence between correlation strength and bridge gain, and an action-based ensemble extension. These constructions provide a reproducible mathematical language for describing when strongly coupled biomedical sectors may become effectively close in disease-state geometry without assuming anatomical continuity or direct causal pathways.

In proof-of-concept simulations on weighted modular graph ensembles, increasing the biomedical correlation proxy systematically reduced effective inter-sector distance and increased bridge gain and fractional compression. The strongest accessibility compression occurred when the correlation-dependent modulation acted on the designated bridge architecture. This bridge-specific effect exceeded the changes observed under no-coupling, random non-bridge modulation, and generic inter-sector modulation controls, supporting the internal consistency of the proposed correlation–compression mechanism.

The results should be interpreted as theoretical and hypothesis-generating. The framework does not establish anatomical pathways, causal biological mechanisms, diagnostic biomarkers, prognostic rules, or treatment recommendations. Instead, it provides a structured way to prioritize candidate hidden connectivity patterns that may later be tested using independent clinical, imaging, molecular, longitudinal, or experimental evidence.

The practical value of the proposed model lies in its potential use as a translational graph-based framework for radiomics, multimodal prognosis, physiological deterioration, recurrence modeling, treatment-response analysis, and patient-specific biomedical networks. Future work should focus on endogenous estimation of sector-level dependence from real patient data, directed and temporal extensions of the accessibility graph, biological interpretation of candidate bridges, and external validation against clinically meaningful endpoints.

## Figures and Tables

**Table 1:** Conceptual positioning of the proposed accessibility-bridge framework relative to related biomedical graph and network approaches.

Approach	Usual Object of Analysis	How Connectivity Is Represented	Relation to the Present Framework	Main Distinction of the Present Work
Network medicine	Genes, proteins, pathways, disease modules, interactomes	Known or inferred biological interactions, disease modules, molecular proximity	Shares the idea that disease is network-organized	Connectivity is not assumed from an interactome; it is inferred as accessibility compression between biomedical sectors
Multilayer biomedical networks	Multiple biological or clinical layers, such as molecular, imaging, clinical, or temporal layers	Inter-layer and intra-layer edges encode cross-domain relations	Compatible with multimodal and multiscale disease organization	Focuses specifically on when inter-sector dependence becomes a localized high-gain accessibility bridge
Radiomics-based graph representations	Imaging features, regions, voxels, habitats, tumor subregions	Spatial adjacency, feature similarity, texture co-variation, or radiomic association	Provides a natural application domain for sector and motif definitions	Does not only describe image-feature similarity; it tests whether radiomic dependence produces effective disease-state distance compression
Information-theoretic approaches	Entropy, mutual information, dependency, uncertainty, information flow	Statistical dependence among variables or subsystems	Mutual-information-type quantities provide the correlation-side observable	The novelty is coupling dependence to graph accessibility geometry and bridge gain
Graph neural networks and graph-based clinical prediction	Patient graphs, EHR graphs, molecular graphs, imaging graphs	Learned edge representations and predictive embeddings	Related to graph-based biomedical representation learning	The present model is not primarily predictive; it provides an interpretable bridge observable before supervised learning
Information-geometric approaches	Statistical manifolds, distributions, divergence measures	Geometry arises from probability distributions or statistical distances	Shares the idea that geometry can emerge from informational structure	The present model defines geometry through constrained graph accessibility and localized bridge compression
Present accessibility-bridge framework	Biomedical sectors, motifs, graph constraints, inter-sector dependence	Localized high-gain reduction in effective accessibility distance	Integrates correlation, graph distance, and bridge observables	Defines hidden connectivity as correlation-induced accessibility compression, not as anatomical continuity or causal proof

**Table 2:** Representative biomedical interpretations of graph nodes and constraint costs in different medical modeling contexts.

Biomedical Context	Possible Nodes *V*	Possible Edge Cost *C*(*e*)
Radiomics/imaging	Tumor regions, peritumoral tissue, edema, necrosis, imaging-derived features	Feature dissimilarity, weak co-variation, reduced spatial or functional accessibility
ICU/physiological modeling	Blood-gas variables, oxygenation indices, inflammatory markers, neurological or outcome variables	Transition difficulty, weak statistical dependence, physiological resistance, reduced predictive accessibility
Oncology/tumor progression	Primary tumor features, immune components, vascular interface, metastatic niche, treatment-response phenotypes	Pathway distance, reduced biological compatibility, weak pathological coupling
Systems medicine	Biomarker modules, molecular pathways, organ systems, disease-state modules	Regulatory separation, reduced information flow, low probability of coordinated state transition

**Table 3:** Main notation used in the biomedical relational graph framework and its corresponding interpretation.

Symbol	Formal Meaning	Biomedical Interpretation
$G=\left(V,E,C\right)$	Biomedical relational configuration	Weighted graph representing clinically relevant entities and their constrained relations
V	Set of graph nodes	Imaging features, tissue regions, biomarkers, physiological variables, disease states
E	Set of admissible relations	Possible biomedical, statistical, functional, or pathological links
*C*(*e*)	Nonnegative constraint cost on relation (e)	Resistance, dissimilarity, weak coupling, reduced accessibility, or transition difficulty
*d*(*i*,*j*)	Node-level accessibility distance	Effective relational separation between two biomedical entities
*d_s_*(*i*,*j*)	Symmetrized accessibility distance	Bidirectional effective separation when directed effects are not emphasized
Ω	Admissible biomedical configuration space	Ensemble of possible disease-network configurations
*S*(*G*)	Informational action/global constraint tension	Overall cost or tension of a biomedical graph configuration
*P*(*G*|*θ*)	Gibbs-type ensemble over configurations	Probability distribution over possible biomedical graph states
*A*,*B* ⊆ *V*	Coarse-grained biomedical sectors	Tumor compartments, organ systems, biomarker modules, physiological domains
*D*(*A*,*B*)	Effective inter-sector accessibility distance	Mean accessibility separation between two biomedical sectors
*x_A_*, *x_B_*	Coarse observables associated with sectors	Radiomic summaries, biomarker activity, physiological instability, motif descriptors
*H_α_*, *H_β_*	Stable biomedical motifs	Persistent disease-relevant patterns or subgraphs supporting sector organization

**Table 4:** Representative simulation parameters used for the proof-of-concept biomedical graph ensemble.

Parameter	Value/Implementation
Number of graph realizations	80
Random seed strategy	master seeds: 101, 202, 303, 404 for the four modulation regimes; visualization seed: 777
Graph type	weighted undirected two-sector modular graph
Total number of nodes	(N = 24)
Number of nodes per sector	12 nodes in sector A and 12 nodes in sector B
Sector node labels	*A*_i_, i = 0, …, 11; *B*_i_, i = 0, …, 11
Internal edge probability	p_intra_ = 0.35 within each sector
Background inter-sector edge probability	p_inter,bg_ = 0.04
Internal connectivity constraint	each sector is forced to be connected by adding missing chain edges
Background inter-sector fallback edges	(A2,B2), (A8,B8), added if absent
Internal edge cost distribution	*C*_0_(*e*) ~ U(0.8,1.6)
Background inter-sector cost distribution	*C*_0_(*e*) ~ U(5.0,8.0)
Planted bridge set $W_{AB}$	(A4,B5), (A5,B4), (A5,B5)
Number of planted bridge edges	3
Planted bridge baseline cost	*C*_0_(*e*) = 1.0 for all e ϵ *W_AB_*
Coupling coefficient	α = 2.5
Biomedical correlation proxy range	$T\left(A:B\right)\epsilon [0,2]$
Number of proxy values	21
Proxy grid	$T\left(A:B\right)$= 0, 0.1, 0.2, …, 2.0
True bridge coupling rule	*C*_AB_(*e*) = *C*_0_(*e*)/(1 + α$T\left(A:B\right)$) for e ϵ *W_AB_*
Non-bridge edges under true coupling	*C*_AB_(*e*) = *C*_0_(e)
Null model 1	no coupling; all edge costs remain *C*_0_(*e*)
Null model 2	random non-bridge modulation; 3 non-bridge edges are randomly selected and modulated
Null model 3	random inter-sector modulation; 3 inter-sector edges are randomly selected and modulated
Background graph for bridge gain	graph with planted bridge edges removed
Accessibility distance	shortest-path cost distance
Shortest-path algorithm	NetworkX single-source Dijkstra path length
Effective inter-sector distance	mean pairwise shortest-path distance *D*(*A*,*B*) over all *a* ϵ *A*, *b* ϵ *B*
Bridge gain	$\Gamma \left(A,B;W_{AB}\right)=D_{bg}\left(A,B;W_{AB}\right)/D(A,B)$
Fractional compression	${∆}_{B}=1-D(A,B)/D_{bg}\left(A,B;W_{AB}\right)$
Reported statistics	mean ± standard deviation across 80 realizations
Software stack	Python 3.12, NumPy 2.0.2, NetworkX, Pandas 2.2.2, Matplotlib 3.10.0
visualization proxy values	$T\left(A:B\right)$= 0.0, 0.8, 2.0

**Table 5:** Principal effect sizes across the four modulation regimes.

Modulation Regime	Δ*D*_eff_	ΔΓ	Δ(Δ_B_)	Interpretation
True bridge coupling	−0.896	+0.723	+0.114	strongest bridge- specific compression
No coupling	0	0	0	negative control
Random non-bridge modulation	−0.316	+0.207	+0.041	weak nonspecific effect
Random inter-sector modulation	−0.694	+0.522	+0.089	intermediate generic cross-sector effect

## Data Availability

No new experimental or clinical data were generated or analyzed in this study. The illustrative numerical simulations used to generate the conceptual figures are based on phenomenological equations described in the manuscript, and the corresponding code can be made available from the corresponding author upon reasonable request.
